# Rapid homeostatic modulation of transsynaptic nanocolumn rings

**DOI:** 10.1073/pnas.2119044119

**Published:** 2022-11-02

**Authors:** Paola Muttathukunnel, Patrick Frei, Sarah Perry, Dion Dickman, Martin Müller

**Affiliations:** ^a^Department of Molecular Life Sciences, University of Zurich, 8057 Zurich, Switzerland;; ^b^Neuroscience Center Zurich, University of Zurich/Swiss Federal Institute of Technology (ETH) Zurich, Zurich, 8057 Switzerland;; ^c^Department of Neurobiology, University of Southern California, Los Angeles, CA 90089

**Keywords:** homeostatic plasticity, synaptic nano-architecture, glutamate receptor, active zone, *Drosophila*

## Abstract

Robust neural information transfer relies on a delicate molecular nano-architecture of chemical synapses. Neurotransmitter release is controlled by a specific arrangement of proteins within presynaptic active zones. How the specific presynaptic molecular architecture relates to postsynaptic organization and how synaptic nano-architecture is transsynaptically regulated to enable stable synaptic transmission remain enigmatic. Using time-gated stimulated emission-depletion microscopy at the *Drosophila* neuromuscular junction, we found that presynaptic nanorings formed by the active-zone scaffold Bruchpilot (Brp) align with postsynaptic glutamate receptor (GluR) rings. Individual rings harbor approximately four transsynaptically aligned Brp-GluR nanocolumns. Similar nanocolumn rings are formed by the presynaptic protein Unc13A and GluRs. Intriguingly, acute GluR impairment triggers transsynaptic nanocolumn formation on the minute timescale during homeostatic plasticity. We reveal distinct phases of structural transsynaptic homeostatic plasticity, with postsynaptic GluR reorganization preceding presynaptic Brp modulation. Finally, homeostatic control of transsynaptic nano-architecture and neurotransmitter release requires the auxiliary GluR subunit Neto. Thus, transsynaptic nanocolumn rings provide a substrate for rapid homeostatic stabilization of synaptic efficacy.

## Significance

Stable neural function is not a given, as apparent from diseases like epilepsy. Robust neural information transfer is especially surprising in light of a remarkable degree of molecular organization of chemical synapses: several synaptic proteins form subsynaptic clusters within individual synapses that align across the synaptic cleft, thereby forming transsynaptic nanocolumns. How transsynaptic nanocolumns are arranged within individual synapses and how they are regulated to enable stable synaptic signaling remain elusive. Here, we discovered transsynaptic nanocolumns composed of presynaptic active-zone proteins and postsynaptic neurotransmitter receptors at *Drosophila* synapses. Intriguingly, these transsynaptic nanocolumns arrange in stereotypic nanorings. Moreover, these transsynaptic nanocolumn rings undergo rapid and defined changes during homeostatic stabilization of synaptic transmission.

Even subtle changes in the molecular architecture of chemical synapses may profoundly affect neural information processing and animal behavior (1, 2). Yet, neural systems are generally stable for a lifetime, implying maintenance of robust synaptic signaling. Stable neural function is especially surprising in light of a remarkable degree of subsynaptic molecular organization of synapses: several proteins locally enrich in subsynaptic clusters within presynaptic active zones (AZs) (2–4), the synaptic cleft (5), and the postsynaptic density (PSD) (6, 7). Moreover, subsynaptic clusters of presynaptic proteins may align with postsynaptic clusters, including neurotransmitter receptor clusters (8–11). These observations gave rise to the concept of transsynaptic nanocolumns. There is some evidence that synaptic transmission predominantly occurs within transsynaptic nanocolumns (8), suggesting that individual synapses may harbor subsynaptic transmission channels. How transsynaptic nanocolumns are arranged within individual synapses is largely unknown.

Several studies discovered that subsynaptic clusters are not randomly distributed within the synaptic compartments (2, 9, 12). For instance, clusters of several presynaptic proteins, including Bruchpilot (Brp; CAST/ELKS) (13), RIM-binding protein (RBP) (2), and Unc13 (4) are organized in stereotypic ring-like arrays within AZs of the *Drosophila* neuromuscular junction (NMJ). These rings are arranged in a key-lock-like fashion at specific distances from a cluster of voltage-gated Ca^2+^ channels at the AZ center (2, 14, 15). This stereotypic topography is thought to specify distinct functional properties of several release sites demarked by Unc13 clusters that are driven by a common Ca^2+^-channel cluster (4, 14). Perturbations of this organization were shown to have profound effects on synaptic transmission and animal behavior (2, 4, 13). Nevertheless, it is unclear how the specific molecular organization of presynaptic AZs relates to postsynaptic architecture.

Compared with the specific nano-organization of presynaptic AZs, knowledge of a corresponding postsynaptic organization is scant. There is evidence for a nonhomogenous and segregated distribution of AMPA and NMDA receptor clusters with regard to the PSD center of mammalian central nervous system (CNS) synapses (6, 7). Recent findings also imply a specific, ring-shaped glutamate receptor (GluR) nano-organization at the *Drosophila* NMJ (16). Yet, the relationship between the arrangement of postsynaptic clusters and presynaptic nano-architecture remains enigmatic.

Synaptic transmission is stabilized by homeostatic modulation of neurotransmitter release (17) and neurotransmitter receptors (18). Despite considerable progress in identifying mechanisms underlying homeostatic regulation of synaptic function (17, 18), comparably little is known about how the molecular organization of synapses is regulated during homeostatic plasticity. At the *Drosophila* NMJ, acute pharmacological or sustained genetic GluR impairment induces an increase in neurotransmitter release that precisely offsets the perturbation (19, 20). Interestingly, two studies suggested the modulation of subsynaptic AZ organization during this form of homeostatic plasticity (21, 22). In particular, acute or sustained GluR perturbation increases the number of subsynaptic Brp, RBP, and Unc13A clusters (22). Whether homeostatic plasticity involves coordinated modulation of synaptic nano-organization across the synaptic compartments is unknown.

Here, we investigate transsynaptic nano-architecture under baseline conditions and during homeostatic plasticity upon GluR perturbation at the *Drosophila* NMJ, using stimulated emission depletion microscopy with time-gated fluorescence detection (gSTED).

## Results

### Transsynaptic Nanocolumn Rings at the *Drosophila* NMJ.

**Fig. 1.**
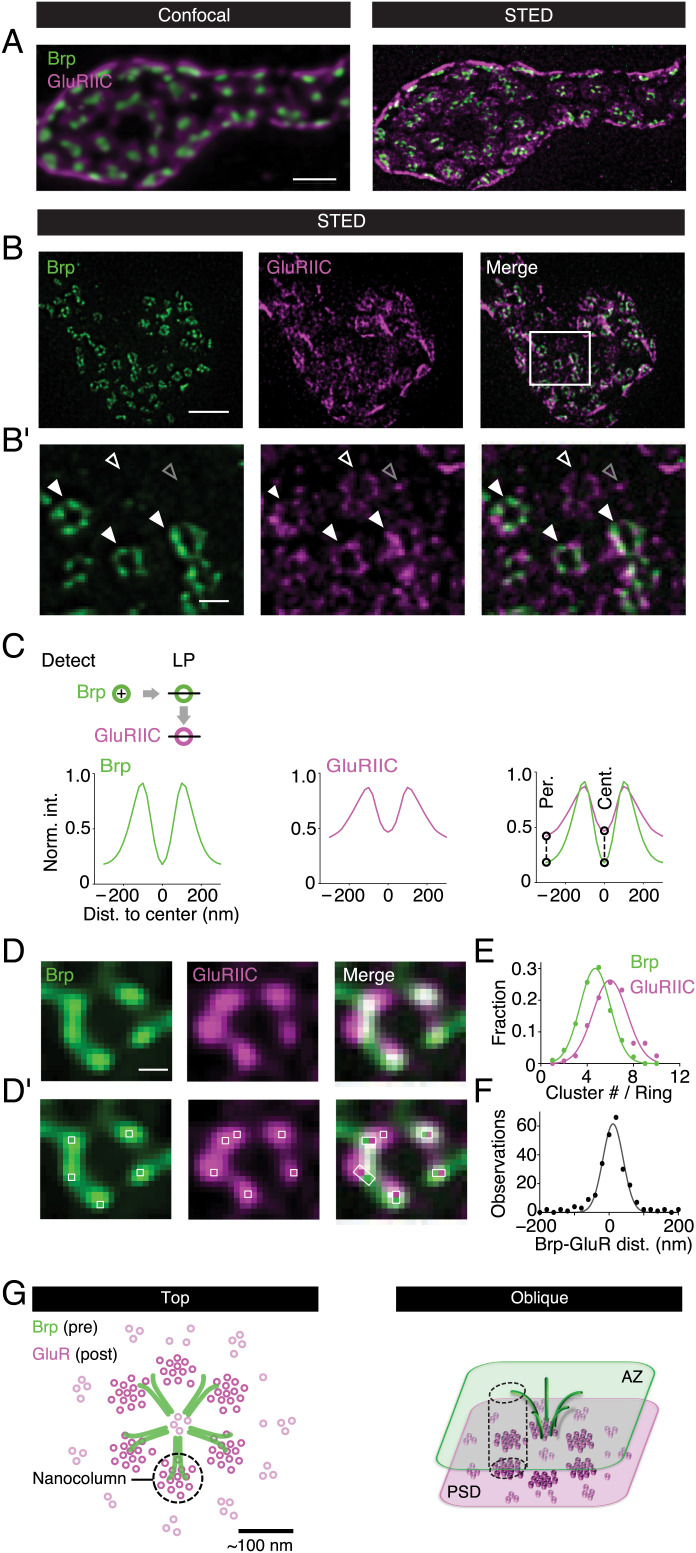
Transsynaptic nanocolumn rings at the *Drosophila* NMJ. (*A*) Maximum intensity projection of synaptic boutons of a wild-type (*w^1118^*) *Drosophila* NMJ (muscle 6) stained with anti-Brp (nc82; green) (13) and anti-GluRIIC (magenta) at confocal (*Left*) and STED resolution (*Right*). (*B* and *B, i*) Synaptic bouton (*B*) and a magnified region (*B, i*, see box in *B*) stained with anti-Brp (green) and anti-GluRIIC (magenta). Filled and empty white arrowheads indicate GluR rings opposed and not opposed by presynaptic Brp rings, respectively. Empty gray arrowhead designates ambient GluR fluorescence spots. (*C*) Schematic of ring detection and line profile (LP) analysis of Brp and opposed GluRIIC rings. Dashed lines indicate quantification at the ring center (Cent.) and periphery (Per.) (*Top*); average Brp and GluR fluorescence intensity LPs normalized to the respective peak obtained after Brp ring detection and subsequent analysis of LPs in the Brp and the GluR channel (*Bottom*; *SI Appendix*) (*n* = 703). Fraction of nonring-like profiles (NRLs, *SI Appendix*, Fig. S*2F*): Brp = 0; GluRIIC = 0.29. (*D* and *D, i*) Higher magnification of Brp (green) and GluRIIC rings (magenta) with local maxima within the rings (bright pixels and white squares, *Bottom*). The white boxes in the merged images illustrate the nearest-neighbor Brp–GluRIIC distance (*Right*). (*E*) Normalized histogram of local maxima number per Brp and GluRIIC ring (Cluster #/Ring) and Gaussian fits (Brp: *n* = 191; GluRIIC: *n* = 125; *P* < 0.0001). (*F*) Histogram of nearest-neighbor distance between Brp and GluR local maxima and Gaussian fit (*SI Appendix*) (*n* = 256). (*G*) Model of subsynaptic GluR organization and Brp–GluRIIC transsynaptic alignment from top (*Left*) and oblique (*Right*) perspective. Scale bars: (*A*) 1 µm; (*B*) 1 µm; (*B, i*) 200 nm; (*D*) 100 nm. Dist, distance; Norm. int., normalized intensity.

Here, we explore subsynaptic molecular organization at the *Drosophila* NMJ employing gSTED microscopy with an effective lateral resolution of <40 nm after image deconvolution (*SI Appendix*, Fig. S1 and *SI Appendix, Materials and Methods*) (23). To study transsynaptic organization at the nanometer scale, we imaged the presynaptic AZ protein Brp together with postsynaptic GluRs (Fig. 1*A*). At the *Drosophila* NMJ, Brp C termini form subsynaptic ring patterns at STED resolution when oriented parallel to the imaging plane (13, 24) (Fig. 1 *B* and *B*, *i*), thereby providing a proxy for synapse orientation. While confocal data did not suggest any specific GluR distribution opposite to presynaptic AZs (Fig. 1*A*) (25), gSTED imaging revealed a distinct distribution of antibodies detecting the essential GluR subunit GluRIIC (Fig. 1 *B* and *B*, *i*). In particular, we observed discrete anti-GluRIIC “spots” (Fig. 1*B*, *i*, gray open arrowhead). A substantial fraction of GluRIIC spots appeared as ring-like patterns (Fig. 1*B*, *i*, white arrowheads), similar to recent observations (16). Intriguingly, these GluRIIC rings were located in close proximity to presynaptic Brp rings (Fig. 1*B*, *i*, filled arrowheads). To analyze GluR distribution and its relationship to Brp, we developed an algorithm for automated ring detection (*SI Appendix, Materials and Methods* and *SI Appendix*, Fig. S2 *A–C*). When detecting Brp and GluR rings independently, the probability of detecting a GluR ring within ≤40 nm of a corresponding Brp-ring center was 0.84 (*n =* 89 rings; *SI Appendix*, Fig. S2 *D* and *E*). We also noted a considerable fraction (22% ± 1%; *n =* 16 NMJs) of ring-shaped GluR fluorescence that was not opposed by presynaptic Brp fluorescence (Fig. 1*B*, *i*, white open arrowheads; *SI Appendix*, Fig. S2*E*). Furthermore, ∼45% of the anti-GluR spots neither aligned with Brp nor formed obvious patterns (Fig. 1*B*, *i*, gray open arrowhead; *SI Appendix*, Fig. S6*K*). We conclude that 1) most Brp rings are opposed by GluR rings, 2) GluR rings can exist without a corresponding Brp ring, and 3) a significant fraction of anti-GluR spots neither align with Brp C termini nor form discernable patterns.

**Fig. 2.**
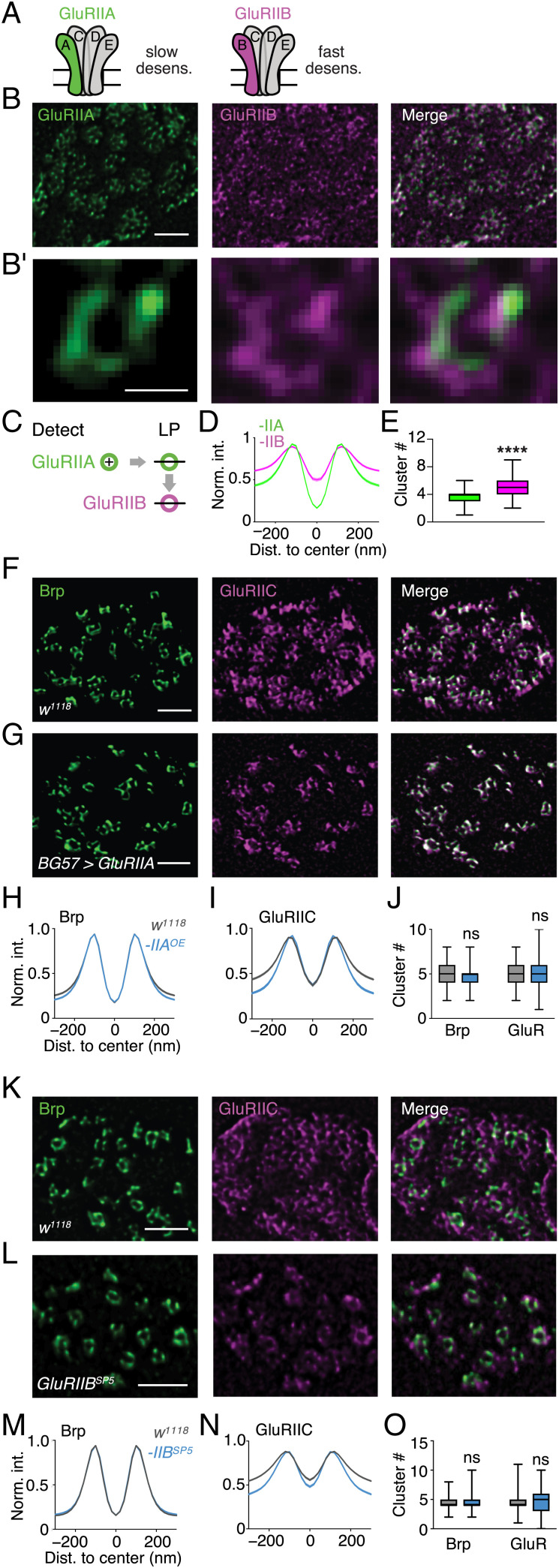
GluR subtype–specific nano-organization. (*A*) Schematic of *Drosophila* GluRIIA and GluRIIB receptor types with slow and fast desensitization (desens). (*B*) Representative anti-GluRIIA (green) and anti-GluRIIB (magenta) staining of a wild-type (*w^1118^*) bouton. (*B, i*) Higher magnification of a GluRIIA (*Left*) and opposed GluRIIB (*Middle*) ring. (*C*) Schematic of GluRIIA ring detection and line-profile (LP) analysis of GluRIIA and opposed GluRIIB rings. (*D* and *E*) Normalized fluorescence intensity (Norm. int.) LPs of the GluRIIA and GluRIIB channel (the shaded area represents SEM), and cluster number (median ± minimum and maximum) within GluRIIA and GluRIIB rings. GluRIIA diameter, mean ± SEM: 243 ± 7 nm, *n =* 204; GluRIIB diameter: 266 ± 7nm, *n =* 165; *P =* 0.0054. (*D*: *n* = 204; *E*: *n* = 176; GluRIIA: 3.619 ± 0.081; GluRIIB: 4.727 ± 0.102; *P* < 0.0001). Nonring-like (NRL) fraction: GluRIIA = 0; GluRIIB = 0.19. (*F* and *G*) Representative example boutons of *w^1118^* and *BG57-Gal4 > UAS-GluRIIA (GluRIIA* OE) stained with anti-Brp (green) and anti-GluRIIC (magenta). (*H*–*J*) Normalized intensity LPs and average cluster numbers of Brp and GluRIIC in *w^1118^* (gray) and *GluRIIA* OE (blue). GluRIIC diameter: *w^1118^*: 248 ± 5 nm, *n* = 266; *GluRIIA* OE: 222 ± 3 nm, *n* = 256; *P* < 0.0001; Brp diameter: w*^1118^*: 222 ± 3 nm, *n* = 313; *GluRIIA* OE: 217 ± 3 nm, *n* = 282; *P* < 0.0001. (*H* and *I*) *w^1118^*: *n* = 247; *GluRIIA* OE: *n* = 282; *J*: Brp: *w^1118^*: 4.826 ± 0.072; *GluRIIA* OE: 4.684 ± 0.064; *P* = 0.258; GluRIIC: *w^1118^*: 5.259 ± 0.089; *GluRIIA* OE: 5.145 ± 0.074; *P* = 0.769). NRL fraction: Brp = 0; GluRIIC: *w^1118^* = 0.15; *GluRIIA* OE = 0.09. (*K* and *L*) Representative example boutons of *w^1118^* and *GluRIIB^SP5^* stained with anti-Brp (green) and anti-GluRIIC (magenta). (*M*–*O*) Normalized intensity LPs and average cluster numbers of Brp and GluRIIC in *w^1118^* (gray) and *GluRIIB^SP5^* (blue). GluRIIC diameter: *w^1118^*: 265 ± 5 nm, *n =* 274; *GluRIIB^SP5^*: 239 ± 4 nm, *n* = 442; *P* < 0.0001; diameter Brp: w*^1118^*: 214 ± 1.9 nm, *n* = 374; *GluRIIB^SP5^*: 211 ± 2 nm, *n* = 573; *P* = 0.26. (*M* and *N*: *w^1118^: n* = 374; *GluRIIB^SP5^: n* = 573; *O*: Brp: *w^1118^*: 4.428 ± 0.049; *GluRIIB^SP5^*: 4.489 ± 0.043; *P* = 0.906; GluRIIC: *w^1118^*: 4.451 ± 0.072; *GluRIIB^SP5^*: 4.543 ± 0.073; *P* = 0.99). NRL fraction: Brp = 0; GluRIIC: *w^1118^* = 0.27; *GluRIIB^SP5^* = 0.23. Scale bars: (*B*) 1 µm; (*B, i*) 200 nm; (*F*) 1 µm; (*G*) 1 µm; (*K*) 1 µm; (*L*) 1 µm. Dist, distance; ns, not significant; **** *P* ≤ 0.0001.

**Fig. 3.**
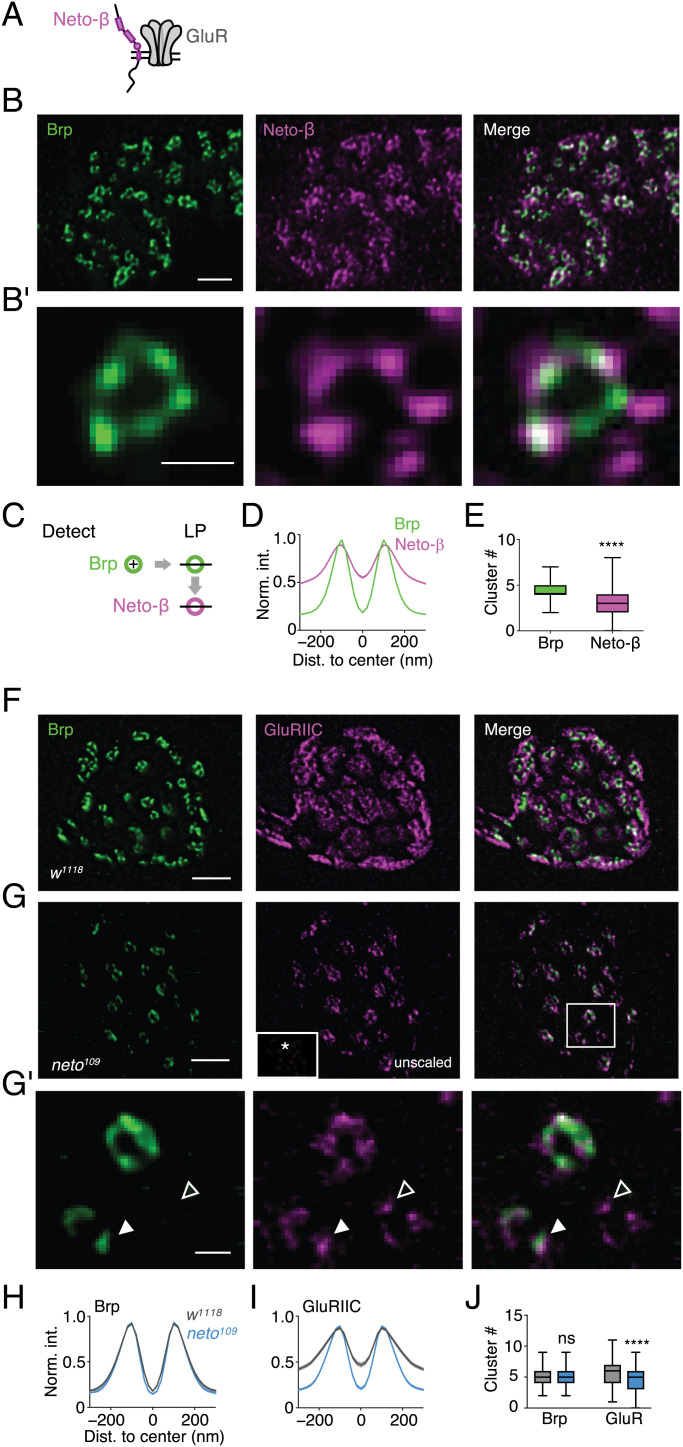
Transsynaptically aligned Neto-β rings stabilize GluRs. (*A*) Schematic of Neto-β and a GluR at the *Drosophila* NMJ. (*B*) Representative anti-Brp (green) and anti–Neto-β staining of a wild-type (*w^1118^*) bouton. (*B, i*) Higher magnification of a Brp (*Left*) and corresponding Neto-β (*Middle*) ring. (*C*) Schematic of Brp ring detection and line-profile (LP) analysis of Brp and opposed Neto-β rings. (*D*) Normalized fluorescence intensity (Norm. int.) LPs of the Brp and the Neto-β channels (*n* = 397; the shaded area represents the SEM). Nonring-like (NRL) fraction: Brp = 0; Neto-β: = 0.27. (*E*) Average cluster number (median ± minimum or maximum) within Brp and Neto-β rings. Brp diameter (mean ± SEM): 209.2 ± 2 nm, *n* = 397; Neto-β diameter: 241 ± 4 nm, *n* = 292; *P* < 0.0001. (Brp: *n* = 325; 4.446 ± 0.966; Neto-β: 3.308 ± 1.558; *P* < 0.0001) (*F* and *G*) Representative example boutons of *w^1118^* and *neto^109^* stained with anti-Brp (green) and anti-GluRIIC (magenta). GluRIIC fluorescence intensity of *neto^109^* (*G*, *Middle*, white box, *same intensity scale as *w^1118^*) was scaled up (*G*, *Middle*). (*G, i*) Higher magnification of *neto^109^* Brp (green) and opposed GluRIIC (magenta) rings. The filled and open arrowheads highlight transsynaptically aligned and unaligned GluR clusters, respectively. (*H*–*J*) Normalized intensity LPs and average cluster number of Brp and GluRIIC in *w^1118^* (gray) and *neto*^109^ (blue). GluRIIC diameter: *w^1118^*: 245 ± 9 nm, *n* = 103; *neto*^109^: 217 ± 4 nm, *n* = 143; *P* = 0.02; Brp diameter: *w^1118^*: 216 ± 4nm, *n* = 133; *neto*^109^: 217 ± 3 nm, *n* = 149; *P* = 0.77. (*H* and *I*: *w^1118^*: *n* = 133; *neto^109^*: *n* = 149; *J*: Brp: *w^1118^*: *n* = 120; 5.258 ± 0.127; *neto^109^*: *n* = 144; 5.236 ± 0.111; *P* > 0.999; GluRIIC: *w^1118^*: *n* = 120; 5.817 ± 0.210; *neto^109^*: *n =* 144; 4.319 ± 0.158; *P* < 0.0001). NRL fraction: Brp = 0; GluRIIC: *w^1118^* = 0.23; *neto^109^* = 0.04. Scale bars: (*B*) 1 µm; (*B, i*) 200 nm; (*F*) 1 µm; (*G*) 1 µm; (*G, i*) 200 nm. Dist, distance; ns, not significant; **** *P* ≤ 0.0001.

We next analyzed the dimensions and transsynaptic alignment of Brp and GluR rings by anchoring line profiles in Brp-ring centers and quantifying the normalized fluorescence intensity of both channels (*SI Appendix, Materials and Methods* and *SI Appendix*, Fig. S2*D*). Remarkably, there was no offset between the Brp and GluRIIC line profiles, indicating tight transsynaptic alignment (Fig. 1*C*, *Right*). Quantifying the interpeak distance of the line profiles revealed similar ring dimensions, with slightly smaller Brp than GluRIIC rings (Brp: 212 ± 5 nm, *n =* 703; GluRIIC: 244 ± 8 nm, *n* = 500; *P =* 0.001; Fig. 1*C*). We also noticed significantly higher relative fluorescence intensities at the ring periphery and center in the GluRIIC vs. the Brp channel, as quantified by comparing relative line-profile intensities at 300 nm and 0 nm distance to the ring center (both *P <* 0.001; Fig. 1*C*, *Right*, dashed lines). This suggests higher GluRIIC background fluorescence and/or a lower density of unaligned GluRs in the ring center and periphery (see below; Figs. 2 and 3). These data demonstrate transsynaptic alignment between Brp and GluR rings of similar dimensions.

Within Brp and GluR rings, fluorescence intensity was heterogeneously distributed (Fig. 1*D*). Earlier work revealed approximately five to six local fluorescence intensity maxima per Brp ring at STED resolution (26), implying that Brp rings are composed of approximately five to six distinct Brp clusters (Fig. 1 *D* and *D*, *i*). To analyze fluorescence within the rings, we developed an algorithm for local fluorescence intensity maximum detection (*SI Appendix, Materials and Methods*, Fig. 1*D*, *i*, and *SI Appendix*, Fig. S3 *A* and *B*). This analysis gave an average number of 4.7 ± 0.1 local maxima per Brp ring (*n =* 191; Fig. 1*D*, *i*, white squares; Fig. 1*E*), and 6.0 ± 0.1 local maxima per GluRIIC ring (*n =* 125; Fig. 1 *D*, *i*, and *E*). Previous direct stochastic optical reconstruction microscopy data suggest that the local maxima within the Brp ring at STED resolution correspond to Brp clusters (fluorescence intensity maxima) or filament bundles, each consisting of ∼30 Brp molecules (27). Correspondingly, the local maxima in the GluR channel likely reflect GluR clusters, similar to findings at mammalian synapses (6, 7, 28). These observations are consistent with the idea that Brp and GluR rings are composed of approximately five and six clusters, respectively.

Next, we explored the relationship between Brp and GluR fluorescence intensity maxima, henceforth called clusters, within the rings by quantifying their lateral nearest-neighbor distance. This revealed an average lateral nearest-neighbor distance of 14 ± 5 nm between Brp and GluRIIC clusters (*n =* 256; Fig. 1*F*), below our lateral resolution (*SI Appendix*, Fig. S1). Line-profile analysis along the Brp-ring circumference revealed that 77% ± 3.4% of the Brp clusters tightly align with GluRIIC clusters (*n* = 30; *SI Appendix*, Fig. S3*C*). Similar results were obtained by manually scoring of transsynaptic Brp–GluR alignment (*SI Appendix*, Fig. S4 *E* and *F*), suggesting that most Brp clusters are opposed by a GluR cluster. In addition to aligned Brp–GluR clusters, we also observed approximately two to three unaligned GluRIIC clusters within the rings (Fig. 1*D* and *SI Appendix*, Fig. S3*C*). Taken together, our experiments uncovered transsynaptically aligned Brp–GluR rings composed of approximately four tightly aligned Brp and GluR clusters, as well as approximately two unaligned GluR clusters. Previous studies termed transsynaptically aligned synaptic protein clusters *transsynaptic nanocolumns* (8, 10). Based on this terminology, our data support a model of transsynaptic nanocolumn rings (Fig. 1*G*). Analysis of synapses likely oriented perpendicular to the imaging plane revealed a concomitant decrease in fluorescence intensity around the center of elongated, transsynaptically aligned Brp–GluRIIC structures (*SI Appendix*, Fig. S4 *G*–*I*). The concomitant intensity decrease in both channels most likely represents aligned “holes” of tilted Brp and GluR rings, further substantiating a model of transsynaptically aligned nanocolumn rings. Furthermore, similar nanocolumn rings were formed by the presynaptic protein Unc13A and GluRs, with approximately three Unc13A clusters per ring, of which two were aligned with GluR and Brp (*SI Appendix*, Fig. S4 *A*–*F*). Earlier work demonstrated that a Ca^2+^-channel cluster localizes to the Brp-ring center (2). Thus, the stereotypic ring topography likely reflects the organization of transsynaptic nanocolumns around a central Ca^2+^-channel cluster that triggers release in response to synaptic stimulation (*Discussion*).

### GluR Subtype-Specific Nano-organization.

While GluR density is highest opposite to Brp C termini (Fig. 1*C*), a significant fraction of anti-GluRIIC fluorescence was detected at the center and outside of the rings (Fig. 1*B*, *i*, open gray arrowhead; Fig. 1*C*). At confocal resolution, ∼60% of the integrated GluRIIC fluorescence intensity was found opposite to Brp (*SI Appendix*, Fig. S4*J*). Similarly, more than half of the GluRIIC clusters localized within the rings at STED resolution (*SI Appendix*, Fig. S6*K*). We next aimed at differentiating between transsynaptically aligned and unaligned GluRs. *Drosophila* GluRs are heterotetramers composed of three essential subunits (GluRIIC, GluRIID, and GluRIIE) and either a GluRIIA or a GluRIIB subunit that determine receptor desensitization (29, 30) (Fig. 2*A*). We therefore hypothesized that subsynaptic GluR organization may be GluR-subtype specific and investigated GluRIIA and GluRIIB distribution with STED microscopy. Analysis of anti-GluRIIA and anti-GluRIIB costaining revealed ring-like arrays of GluRIIA and GluRIIB fluorescence of similar dimensions (Fig. 2 *B*–*D*), suggesting that receptors within the rings incorporate both the GluRIIA and the GluRIIB subunit. GluRIIA-centered line-profile analysis (Fig. 2*C*) showed significantly higher relative GluRIIB fluorescence intensities toward the ring periphery and in the ring center compared with GluRIIA (GluRIIA: *n* = 204; GluRIIB: *n* = 165; *P* < 0.0001; Fig. 2*D*), either indicating that GluRs outside the rings predominately contain GluRIIB, or a higher background fluorescence intensity in the GluRIIB channel (see below). Within the rings, we detected approximately four or five clusters in the GluRIIA and the GluRIIB channel, respectively (*P* < 0.0001; Fig. 2*E*). As individual rings contain on average approximately five Brp clusters and approximately six GluRIIC clusters (Fig. 1*E*), this implies that most transsynaptic nanocolumns likely harbor both, GluRIIA- and GluRIIB-containing receptors.

While anti-GluRIIA fluorescence was largely confined to the rings, anti-GluRIIB fluorescence was found inside and outside the rings (Fig. 2 *B* and *D*). To test whether the differential anti-GluRIIA and anti-GluRIIB fluorescence distribution is due to a GluR subtype–specific nano-organization, we assayed GluR organization after genetic manipulation of these two subunits. GluRIIA-containing GluRs primarily localize to rings (Fig. 2 *B* and *D*). We thus hypothesized that GluR rings are more distinct upon *GluRIIA* overexpression. First, GluRIIB fluorescence intensity and area was reduced upon postsynaptic *GluRIIA* overexpression (*BG57-Gal4* > *UAS-GluRIIA*) at confocal resolution (*SI Appendix*, Fig. S6 *F*–*H*), suggesting a redistribution toward GluRIIA-containing receptors. At STED resolution, GluRIIC fluorescence appeared more distinct and ring-like after *GluRIIA* overexpression, compared with wild-type (*w^1118^*) *Drosophila* synapses (Fig. 2 *F* and *G*, *Middle*). Line-profile analysis of aligned Brp–GluRIIC rings revealed significantly lower relative GluRIIC fluorescence intensity at the ring periphery, but not in the ring center, upon GluRIIA overexpression (*w^1118^*: *n* = 268; *BG57-Gal4* > *UAS-GluRIIA*: *n* = 257; periphery: *P* < 0.0001; center: *P* > 0.99; Fig. 2*I* and *SI Appendix*, Fig. S6*J*), implying more distinct GluR rings due to reduced GluR density in the ring periphery. The reduction in GluR fluorescence outside the rings indicates the existence of GluRs that are not aligned to Brp, which are henceforth called ambient GluRs. Intriguingly, while GluRIIC rings were slightly broader than Brp rings in the wild type (Fig. 1*C* and *SI Appendix*, Fig. S2*F,* S4*D* and S6*B*), there was no offset between the peaks of GluRIIC and Brp line profiles after *GluRIIA* overexpression (*SI Appendix*, Fig. S6 *A* and *B* and *L* and *M*), suggesting tight transsynaptic alignment between the rings. We did not observe significant changes in cluster number within Brp and GluRIIC rings between the two genotypes (Brp: *P* = 0.26; GluRIIC: *P* = 0.77; Fig. 2*J*), implying that *GluRIIA* overexpression does not affect cluster number within the ring. However, *GluRIIA* overexpression decreased the fraction of GluRIIC clusters outside the rings (*SI Appendix*, Fig. S6*K*) and increased the percentage of Brp-aligned GluRIIC clusters (*SI Appendix*, Fig. S6*I*). Thus, GluRIIA predominately localizes to transsynaptic nanocolumn rings, and *GluRIIA* overexpression results in tight transsynaptic alignment between Brp and GluR nanorings.

Anti-GluRIIB fluorescence outside the rings indicates that ambient receptors may preferentially incorporate the GluRIIB subunit (Fig. 2 *B* and *D*). Loss of the GluRIIB subunit is therefore expected to decrease anti-GluRIIC levels outside the rings and to result in more distinct GluR rings. We therefore used CRISPR/Cas9–targeted mutagenesis to generate null mutations in the *GluRIIB* locus [*GluRIIB^SP^*^5^; *Materials and Methods* (31)]. Anti-GluRIIA fluorescence intensity was increased, whereas anti-GluRIIB fluorescence intensity was strongly decreased, in *GluRIIB^SP^*^5^ mutants (*SI Appendix*, Fig. S7 *A*–*F*), implying that GluRIIB-containing receptors were replaced by GluRIIA-containing receptors. *GluRIIB^SP^*^5^ mutants displayed distinct GluRIIC rings (Fig. 2 *K* and *L*), and relative GluRIIC line profile intensity was significantly dimmer toward the ring periphery and in the ring center in *GluRIIB^SP^*^5^ mutants compared with wild type (*w^1118^*: *n* = 374; *GluRIIB^SP5^*: *n* = 573; periphery: *P* < 0.0001; center: *P* = 0.001; Fig. 2*N*), suggesting the loss of ambient GluRs. Brp line profiles (Fig. 2*M*), as well as Brp and GluRIIC cluster numbers, were similar between *GluRIIB^SP5^* and wild type (line profile: periphery: *P* = 0.84; center: *P* > 0.99; cluster: *w^1118^*: *n* = 374; *GluRIIB^SP5^*: *n* = 573; Brp: *P =* 0.91; GluRIIC: *P =* 0.99; Fig. 2 *M*–*O*), indicating that loss of the GluRIIB subunit does not affect Brp dimensions and Brp or GluRIIC nanocolumn number. We conclude that loss of *GluRIIB* leads to a predominant loss of receptors outside the ring and to the replacement of GluRIIA by GluRIIB-containing receptors within the ring. This, in turn, suggests that GluRIIB-containing receptors localize within and outside of transsynaptic nanocolumn rings in wild type. In line with this model, *GluRIIA^SP16^* mutants displayed similar transsynaptic nanocolumn rings as wild type (*SI Appendix*, Fig. S5 *E*–*I*). At the physiological level, we revealed increased miniature excitatory postsynaptic potentials (mEPSP) amplitudes in *GluRIIB^SP5^* mutants compared with controls (*SI Appendix*, Fig. S7*G*), implying that GluRIIB-containing receptors have the potential to negatively regulate synaptic transmission, likely by replacing GluRIIA-containing receptors within the nanocolumns (*SI Appendix*, Fig. S7 *C*–*H*) (*Discussion*). Together, our investigation of GluR subtypes demonstrates that *GluRIIA* overexpression and *GluRIIB* loss result in distinct transsynaptic nanocolumn rings and support a model of GluR subtype–specific nano-organization.

### Transsynaptically Aligned Neto-β Rings Stabilize GluRs.

We next sought to provide independent evidence for transsynaptically aligned rings by analyzing the subsynaptic distribution of Neto, an auxiliary GluR subunit previously suggested to play a role in GluR clustering (32, 33) (Fig. 3*A*). There are two Neto isoforms with different expression patterns at the *Drosophila* NMJ: while Neto-α is expressed both pre- and postsynaptically (34), Neto-β is the major postsynaptic isoform at the *Drosophila* NMJ. Anti–Neto-β is arranged in ring-like arrays in close proximity to presynaptic Brp (Fig. 3 *B* and *B*, *i*). We also observed a significant fraction of anti–Neto-β that was not opposed by Brp (Fig. 3*B*), similar to GluRIIC (Fig. 1 *B* and *B*, *i*). Line-profile analysis revealed an overlap between Brp and Neto-β line-profile peaks (Fig. 3*D*), demonstrating similar dimensions and transsynaptic alignment. Similar to GluRIIC, Neto-β fluorescence intensity was higher at the ring periphery and center compared with Brp (Neto-β: *n* = 149; Brp: *n* = 274; periphery: *P* < 0.0001; center: *P* < 0.0001; Fig. 3*D*), suggesting that Neto-β localizes inside and outside of transsynaptically aligned rings. Neto-β line profiles also aligned with anti-GluRIIA line profiles (*SI Appendix*, Fig. S8 *A* and *B*). The close relationship between Neto-β and GluRIIA rings is in line with genetic data suggesting that Neto-β predominantly stabilizes GluRIIA-containing receptors (33). However, the Neto-β fluorescence outside the rings (Fig. 3 *B*, *i* and *D*) indicates that this auxiliary GluR subunit also interacts with other proteins, the existence of a Neto-β reserve pool, or background fluorescence. Within transsynaptically aligned Neto-β rings, we detected 3.3 ± 1.5 clusters (*n =* 397; Fig. 3*E*), significantly fewer than for Brp (∼5 clusters; *P* < 0.0001; Fig. 1*E*), GluRIIC (∼6 clusters; *P* < 0.0001; Fig. 1*E*), or GluRIIA (∼4 clusters; *P* < 0.0001 *SI Appendix*, Fig. S8*D*), suggesting that Neto-β is unlikely part of every nanocolumn. Furthermore, ∼50% of the Neto-β clusters aligned with GluRIID (*SI Appendix*, Fig. S12*K*). We conclude that Neto-β forms rings that are transsynaptically aligned with Brp and are composed of approximately three clusters. Hence, Neto-β provides independent evidence for postsynaptic nanorings and constitutes another postsynaptic component of transsynaptic nanocolumn rings.

Neto is thought to be required for GluR clustering at the *Drosophila* NMJ (33). We next assessed how loss of *neto* affects subsynaptic organization. GluRIIC fluorescence intensity was strongly decreased in hypomorphic *neto^109^* mutants at confocal resolution (*SI Appendix*, Fig. S9 *A* and *B*), in which Neto-α and Neto-β levels are strongly reduced (33), implying GluR loss, consistent with previous work (32). Furthermore, we noted a marked reduction in GluRIIA and GluRIIB fluorescence intensity in *neto^109^* mutants (*SI Appendix*, Fig. S9 *I*–*M*), suggesting that Neto stabilizes both receptor types. At STED resolution, GluRIIC rings appeared dimmer, and GluRIIC intensity inside and outside the rings was reduced (Fig. 3*G*, *Middle*, inset), suggesting GluR loss inside and outside the rings. As evident from the bouton with scaled GluRIIC fluorescence intensity shown in Fig. 3*G* (*Middle*), the remaining GluRs formed very distinct rings that transsynaptically aligned with Brp rings (Fig. 3 *G* and *G*, *i*). We observed a significant increase in the fraction of ring-like GluRIIC line profiles in *neto^109^* mutants (*SI Appendix*, Fig. S9 *C* and *D*), as well as a significant decrease in normalized fluorescence intensity in the ring periphery and the ring center (*w^1118^*: *n* = 103; *neto^109^*: *n* = 143; periphery and center: *P <* 0.0001; Fig. 3*I*), indicating that the levels of GluRs not aligned to the Brp C-termini were decreased more strongly than aligned GluRs in *neto^109^* mutants. Consequently, while GluRIIC line profiles were broader than Brp line profiles in wild type, GluRIIC and Brp line profiles were very similar and tightly aligned in *neto^109^* mutants (*SI Appendix*, Fig. S9 *E–G*). Together with the decrease in ambient GluR levels upon *GluRIIA* overexpression or in *GluRIIB* mutants (Fig. 2), these data again imply that GluRs reside at the ring periphery and center of wild-type synapses (*SI Appendix*, Fig. S6 *L*–*O*). We also noted a slight but significant decrease in GluRIIC cluster number within the rings of *neto^109^* mutants (*w^1118^*: *n* = 133; *neto^109^*: *n* = 149; *P <* 0.0001; Fig. 3*J*). Note that we did not observe a correlation between cluster count and fluorescence intensity in our data (*SI Appendix*, Fig. S8*D*), indicating that the reduction in GluRIIC cluster number in *neto^109^* is unlikely due to a decrease in fluorescence intensity. The decreased GluR levels in *neto^109^* mutants also led to more distinct GluR clusters within the rings (Fig. 3*G*, *i*, *Middle*, arrowheads), likely caused by a lower GluR abundance. While we observed some GluR clusters within the rings that were not opposed by Brp fluorescence (Fig. 3*G*, *i*, *Middle*, open arrowhead), the fraction of Brp-aligned GluRIIC clusters increased in *neto^109^* mutants (*SI Appendix*, Fig. S9*H* and Fig. 3*G*, *i*, *Middle*, filled arrowhead), indicating that the remaining GluR clusters predominantly localize in close proximity to Brp clusters, consistent with a model of transsynaptic nanocolumn rings. We did not observe apparent changes in Brp intensity (periphery: *P* = 0.196; center: *P* = 0.22), or Brp cluster number between the two genotypes (*w^1118^*: *n* = 133; *neto^109^*: *n* = 149; *P ≥* 0.99; Fig. 3 *H*–*J*). Taken together, the investigation of the auxiliary GluR subunit Neto further corroborates a model of postsynaptic ring patterns that are aligned across the synaptic cleft, and reveals that Neto stabilizes GluRIIA- and GluRIIB-containing receptors inside and outside the nanocolumn rings. To test the specificity of the effects observed after *neto* perturbation, we examined transsynaptic nano-organization at synapses lacking dSol-1, a recently identified auxiliary GluR subunit (35). *dSol-1* null mutant synapses exhibited increased GluR fields and largely unchanged transsynaptic Brp–GluR nano-architecture (*SI Appendix*, Fig. S10), implying that different auxiliary GluR subunits play different roles in shaping transsynaptic nano-architecture.

### Rapid Homeostatic Modulation of Transsynaptic Nanocolumn Rings.

**Fig. 4.**
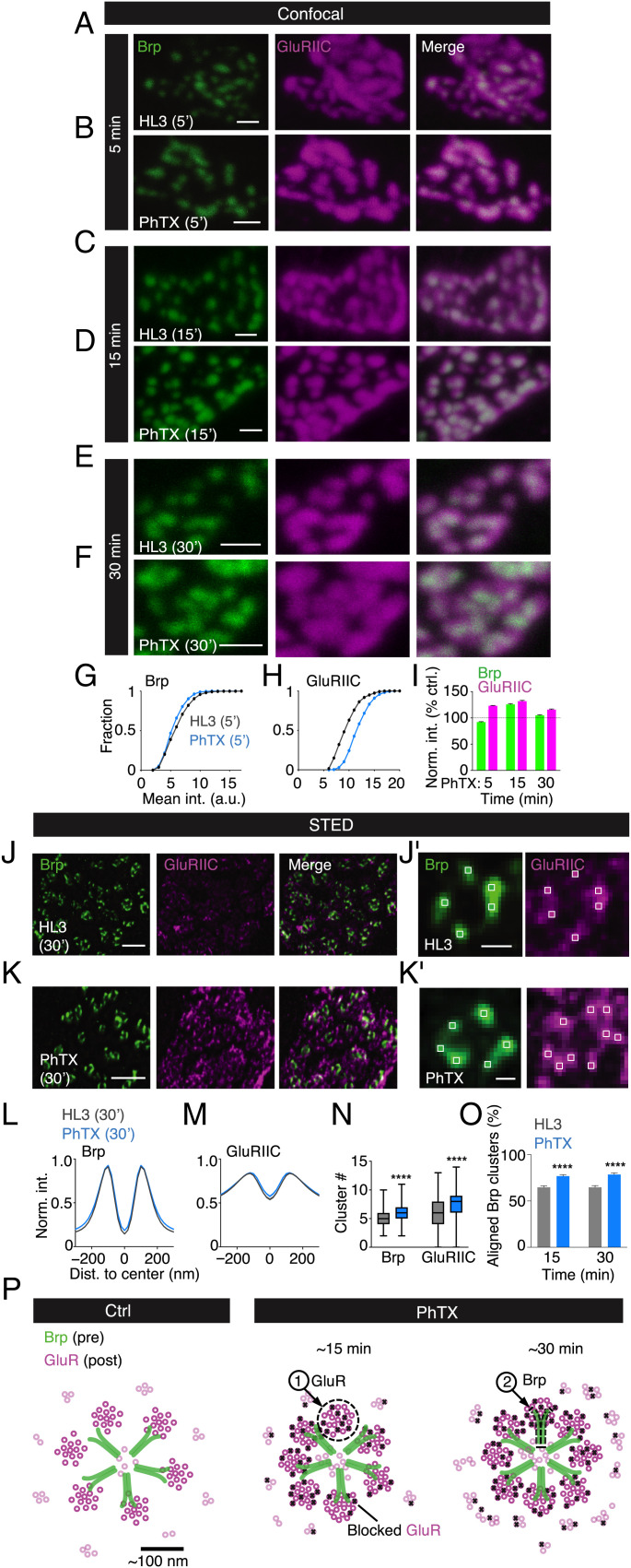
Rapid and sequential modulation of transsynaptic nanocolumn rings during homeostatic plasticity. (*A*–*F*) Representative wild-type (*w^1118^*) boutons stained with anti-Brp and anti-GluRIIC upon HL3/saline or PhTX treatment, both for 5 min (*A* and *B*), 15 min (*C* and *D*), or 30 min (*E* and *F*), at confocal resolution. (*G* and *H*) Corresponding cumulative frequency histogram of mean fluorescence intensity changes of Brp (*Left*) and GluRIIC (*Right*) channels upon HL3/saline (gray) or PhTX (blue) treatment (both for 5 min) (*G*: HL3: *n* = 756; 6.465 ± 0.078; PhTX: *n* = 940; 5.947 ± 0.058; *P* < 0.0001; *H*: HL3: *n* = 777; 9.934 ± 0.085; PhTX: *n* = 1066; 12.22 ± 0.070; *P* < 0.0001). (*I*) Relative changes in Brp and GluRIIC mean intensity upon PhTX incubation for 5, 15, and 30 min normalized to time-matched HL3/saline-treated controls. (Brp: *n* (5′) = 940; 91.98 ± 0.903; *P* < 0.0001; *n* (15’) = 844; 125.6 ± 1.560; *P* < 0.0001; *n* (30’) = 1736; 104.9 ±1.083; *P* < 0.0001; GluRIIC: *n* (5′) = 1066; 123.0 ± 0.705; *P* < 0.0001; *n* (15’) = 724; 131.8 ± 1.590; *P* < 0.0001; *n* (30’) = 1333; 104.9 ±1.083; *P* < 0.0001). (*J* and *K*) Representative *w^1118^* boutons stained with anti-Brp (green) and anti-GluRIIC (magenta) upon HL3 or PhTX treatment (both: 30 min) at STED resolution. (*J, i* and *K, i*) Brp and GluRIIC ring examples with local maxima (white boxes). (*L* and *M*) Corresponding normalized intensity (Norm. int.) line profiles of the Brp and GluRIIC channel upon HL3 (gray) or PhTX (blue) treatment (both for 30 min) (*L* and *M*: HL3: *n* = 703; PhTX*: n* = 706). Nonring-like (NRL) fraction: Brp = 0; GluRIIC: HL3 = 0.28; PhTX = 0.31. (*N*) Average cluster numbers within Brp (green) and GluRIIC (magenta) rings upon HL3 or PhTX treatment (both for 30 min). GluRIIC diameter: HL3: 296 ± 5 nm, *n* = 500; PhTX: 281 ± 5 nm, *n* = 487; *P* = 0.04; Brp diameter: HL3: 217 ± 2, *n* = 703; PhTX: 215 ± 2, *n* = 705; *P* = 0.57. (Brp: HL3: *n* = 670; 4.964 ± 0.048; PhTX: *n* = 705; 5.664 ± 0.058; *P* < 0.0001; GluRIIC: HL3: *n* = 670; 5.775 ± 0.101; PhTX: *n =* 705; 7.487 ± 0.090; *P* < 0.0001). (*O*) Average number of Brp clusters aligned to GluRIIC (%) normalized to the total Brp cluster number within Brp rings following 15-min or 30-min treatment with HL3 (gray) or PhTX (blue) (for quantification, see *SI Appendix*, Fig. S4*E* ). 15 min: HL3: *n* = 158; 64.72% ± 1.56%; PhTX: *n* = 113; 76.76% ± 1.68%; *P* < 0.0001; 30 min: HL3: *n* = 115; 64.67% ± 1.84%; PhTX: *n* = 92; 78.58% ± 1.77%; *P* < 0.0001). (*P*) Model illustrating changes in subsynaptic organization of transsynaptically aligned Brp and GluR rings upon PhTX-induced GluR perturbation. Note that the model does not reflect changes seen at confocal resolution (*SI Appendix*, Fig. S11). Scale bars: (*A*–*F*) 1 µm; (*J*): 1 µm; (*K*) 1 µm; (*J, i*) 100 nm; (*K, i*) 100 nm; (*P*) 100 nm. a.u., arbitrary unit; Dist, distance; int, intensity; ns, not significant; **** *P* ≤ 0.0001.

Having provided evidence for transsynaptic nanocolumn rings, we next asked if they are modulated during synaptic plasticity. At the *Drosophila* NMJ, pharmacological GluR impairment induces a homeostatic increase in neurotransmitter release that precisely compensates for the perturbation within minutes after receptor impairment (20, 21, 36, 37). There is evidence that Brp content per AZ and Brp cluster number is increased within 10 min during this form of homeostatic plasticity, commonly referred to as presynaptic homeostatic plasticity (PHP) (22). Based on the transsynaptic alignment between Brp and GluR rings, we hypothesized a modulation of transsynaptic Brp–GluR nanocolumns during PHP. First, we employed confocal microscopy to probe relative changes in Brp and GluR fluorescence intensity during homeostatic plasticity (Fig. 4 *A*–*F*). Application of the GluR antagonist philanthotoxin-433 (PhTX) for 5 min decreased mean Brp fluorescence intensity compared with saline-treated controls (HL3-saline: *n* = 756; PhTX: *n* = 940; −8.0%; *P <* 0.0001; Fig. 4 *A*, *B*, *G*, *I*), implying a slight decrease in Brp levels. By contrast, we noted a prominent increase in GluRIIC fluorescence intensity after PhTX treatment for 5 min (HL3-saline: *n* = 777; PhTX: *n* = 1,066; +23.0%; *P <* 0.0001; Fig. 4 *A*, *B*, *H*, and *I*), suggesting increased GluR levels. Furthermore, GluR area significantly decreased after 5 min of PhTX application (*SI Appendix*, Fig. S11*A*), implying that the increase in anti-GluR fluorescence intensity may be due to GluR redistribution (*SI Appendix*, Fig. S11 *A* and *B*). After 15 min of PhTX treatment, there was a significant increase in both Brp fluorescence intensity (HL3: *n* = 1,126; PhTX: *n* = 844; +25.6%; *P <* 0.0001; Fig. 4 *C*, *D* and *I*) and GluRIIC fluorescence intensity (HL3: *n* = 1,280; PhTX: *n* = 724; +31.8%; *P <* 0.0001; Fig. 4 *C*, *D* and *I*), compared with untreated controls. Similar results were obtained after PhTX incubation for 30 min (Brp: HL3: *n* = 1,910; PhTX: *n* = 1,736; +5%; *P <* 0.0001; GluRIIC: HL3: *n* = 1,386; PhTX: *n* = 1,333; +16%; *P <* 0.0001; Fig. 4 *E*, *F* and *I*). Note that the magnitude of the relative changes cannot be compared between the different time points, because the data were obtained using different settings (*Materials and Methods*). These data provide evidence that pharmacological GluR perturbation induces a rapid and sequential increase in synaptic GluRIIC and Brp abundance on the minute time scale, with GluRIIC modulation preceding Brp modulation. In addition, Brp and GluRIIC fluorescence intensity was elevated in *GluRIIA^SP16^* mutants, which express chronic PHP upon loss of the GluRIIA subunit (*SI Appendix*, Fig. S5 *A*–*D*) (19). This indicates that GluRs are also modulated during chronic PHP, in line with earlier work (16).

We next used gSTED imaging to investigate transsynaptic architecture during rapid homeostatic plasticity (Fig. 4 *J*–*O*). While we did not detect significant changes in the organization of Brp and GluR rings after PhTX application for 5 min (*SI Appendix*, Fig. S11 *A*–*C*), there was a significant increase in GluR cluster number (HL3: *n* = 500; PhTX: *n* = 487; *P* = 0.008), but no significant change in Brp cluster number (*P =* 0.80) upon PhTX incubation for 15 min (*SI Appendix*, Fig. S11*C*). Furthermore, we noted a significant increase in the fraction of transsynaptically aligned Brp clusters (HL3: *n* = 158; PhTX: *n* = 113; *P <* 0.0001; Fig. 4*O*), indicative of an increase in transsynaptic nanocolumn number. The increase in nanocolumn number without major changes in Brp cluster number implies that the newly formed GluR clusters align with existing Brp clusters. After PhTX exposure for 30 min, both GluRIIC and Brp cluster numbers significantly increased (HL3: *n* = 703; PhTX: *n* = 705; both *P <* 0.0001; Fig. 4 *J*, *i*; *K, i*; and N; *SI Appendix*, Fig. S11*C*), without major changes in GluRIIC and Brp ring diameters (GluRIIC: HL3: *n* = 500; PhTX: *n* = 487; *P =* 0.04; Brp: HL3: *n* = 703; PhTX: *n* = 705; *P =* 0.57; Fig. 4 *L* and *M*). Similar to the 15-min time point, the fraction of transsynaptically aligned Brp clusters increased after 30 min of PhTX treatment (HL3: *n* = 115; PhTX: *n* = 92; *P <* 0.0001; Fig. 4*O*), in agreement with an increase in transsynaptic nanocolumn abundance upon GluR impairment. Thus, in addition to an increase in Brp and GluR cluster number per ring, there is an increased fraction of transsynaptically aligned clusters during PHP. These observations suggest transsynaptically coordinated modulation of synaptic nano-architecture during PHP. Consistent with our confocal data, the increase in GluR cluster number preceded the increase in Brp cluster number, implying sequential modulation of GluR and Brp cluster numbers during PHP. The delay between the changes at confocal and STED resolution may indicate that changes in GluR and Brp distribution and/or levels precede the increase in nanocolumn number (*SI Appendix*, Fig. S11 and *Discussion*). While we observed elevated Brp and GluRIIC fluorescence intensity during chronic PHP in *GluRIIA^SP16^* mutants at confocal resolution (*SI Appendix*, Fig. S5 *A*–*D*), we did not detect apparent changes in cluster number or ring diameter for either Brp or GluRIIC in *GluRIIA^SP16^* mutants with STED microscopy (*SI Appendix*, Fig. S5 *E*–*I*). This either indicates that we could not resolve the changes previously reported for Brp during chronic PHP (22), or that chronic and acute PHP involve differential modulation of transsynaptic nano-architecture. Together, our data suggest rapid, sequential modulation of transsynaptic Brp–GluR nanocolumn rings during homeostatic plasticity upon pharmacological GluR inhibition (Fig. 4*P*).

### Homeostatic Modulation of Transsynaptic Nano-Organization and Presynaptic Release Requires *neto*.

**Fig. 5.**
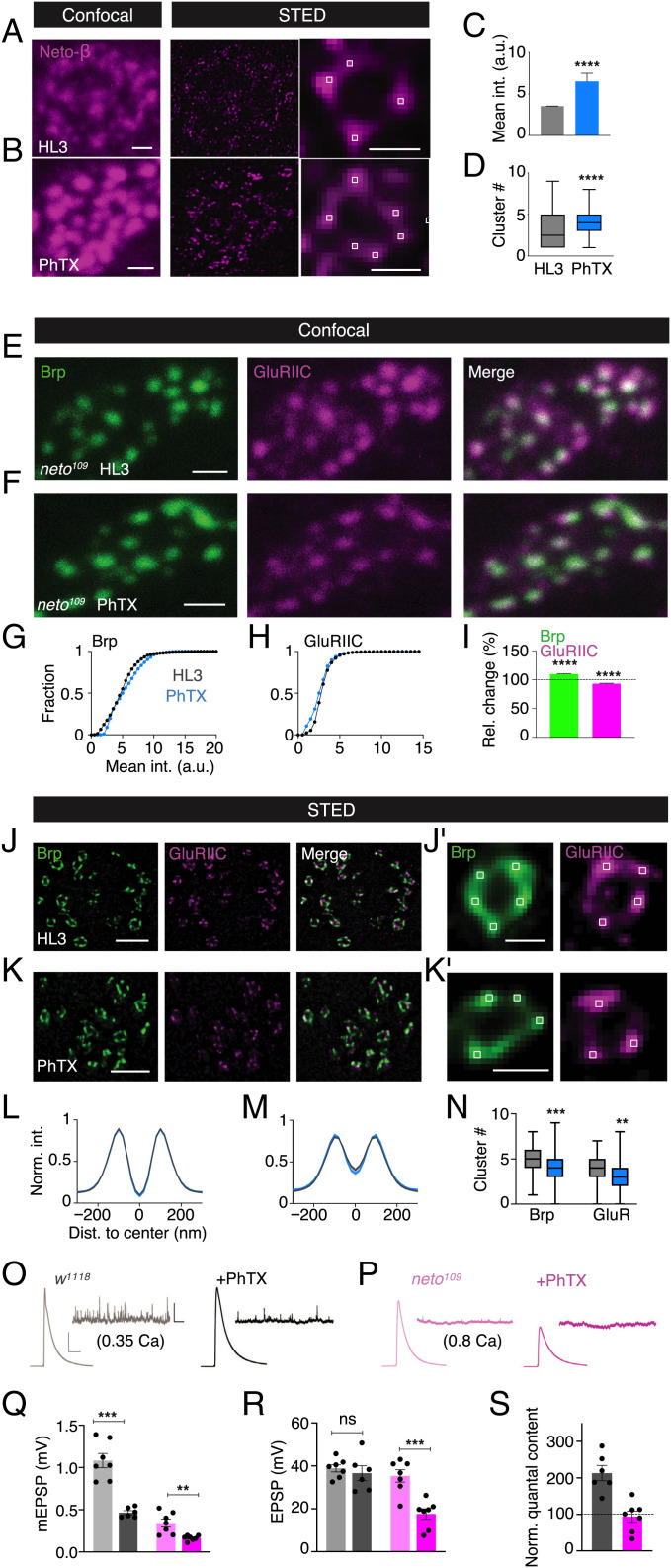
Rapid homeostatic increase in GluR abundance and transsynaptic nanocolumn number requires *neto*. (*A* and *B*) Representative wild-type (*w^1118^*) boutons stained with anti–Neto-β upon HL3/saline or PhTX treatment (both for 15 min) at confocal (*Left*) and STED (*Middle*) resolution. Representative Neto-β rings with corresponding local maxima (white squares) are shown on the *Right*. (*C* and *D*) Corresponding mean intensity (int.) and cluster number quantification of Neto-β upon HL3/saline (gray) or PhTX (blue) (both for 15 min) (*C*: HL3, *n* = 3278; 3.534 ± 0.019; PhTX, *n* = 777; 6.522 ± 0.990; *P* < 0.0001; *D*: HL3, *n* = 268; 3.138 ± 0.125; PhTX, *n* = 4.055 ± 0.103; *P* < 0.0001). (*E* and *F*) Representative *neto^109^* boutons stained with anti-Brp and anti-GluRIIC upon HL3 or PhTX treatment (both for 15 min) at confocal resolution. (*G* and *H*) Corresponding cumulative frequency histograms of mean Brp (*Left*) and GluRIIC fluorescence intensity (*Right*) upon HL3/saline (gray) or PhTX treatment (blue; both 15 min) in *neto^109^*. (*G*: HL3: *n* = 2,162; 5.092 ± 0.053; PhTX: *n* = 1,775; 5.641 ± 0.059; *P* < 0.0001; *H*: HL3: *n* = 2,363; 3.169 ± 0.023; PhTX: *n* = 2,497; 2.839 ± 0.025; *P* < 0.0001). (*I*) Mean Brp and GluRIIC fluorescence intensity after PhTX application (15 min) normalized to time-matched HL3/saline controls (15 min). (Brp: *n* = 1,775; 109.8 ± 0.896; GluRIIC: *n* = 2,497; 93.21 ± 0.679; both: *P* < 0.0001). (*J* and *K*) Representative *neto^109^* boutons stained with anti-Brp and anti-GluRIIC upon HL3 or PhTX treatment (15 min) at STED resolution. (*J, i* and *K, i*) Representative Brp and GluRIIC rings in *neto^109^* incubated with HL3 or PhTX (both for 15 min) with corresponding local maxima (white squares). (*L*–*N*) Corresponding normalized intensity (norm. int.) line profiles and average cluster number of the Brp (green) and GluRIIC channel (magenta) upon HL3 (gray) or PhTX (blue) treatment (both for 15 min) in *neto^109^*. GluRIIC diameter: HL3: 296 ± 5 nm, *n* = 500; PhTX: 281 ± 5 nm, *n* = 487; *P* = 0.04; Brp diameter: HL3: 217 ± 2, *n* = 703; PhTX: 215 ± 2, *n* = 705; *P* = 0.57. (*L* and *M*: HL3, *n* = 332; PhTX, *n* = 295; *N*: Brp: HL3: *n* = 287; 4.610 ± 0.076; PhTX: *n* = 253; 4.103 ± 0.086; *P* = 0.0002; GluRIIC: HL3: *n* = 274; 3.631 ± 0.087; PhTX: *n =* 247; 3.174 ± 0.095; *P* = 0.0012). (*O*) Representative mEPSP and EPSP traces in *w^1118^* upon HL3 (gray) or PhTX (black) treatment (both for 10 min). (*P*) Representative mEPSP and EPSP traces in *neto^109^* upon HL3 (light pink) or PhTX (dark pink) treatment (both for 10 min). (*Q* and *R*) Average mEPSP and EPSP amplitude in *w^1118^* and *neto^109^* upon HL3 or PhTX treatment. (*Q*: *w^1118^*: HL3, *n* = 7; 1.082 ± 0.085; PhTX, *n* = 6; 0.466 ± 0.023; *P* = 0.0003; *neto^109^*: HL3, *n* = 7; 0.342 ± 0.048; PhTX, *n* = 7; 0.166 ± 0.012; *P* = 0.0098; *R*: *w^1118^*: HL3, *n* = 7; 38.87 ± 1.677; PhTX, *n* = 6; 36.62 ± 3.454; *P* = 0.999; *neto^109^*: HL3, *n* = 7; 35.34 ± 2.874; PhTX, *n* = 7; 17.56 ± 2.471; *P* = 0.0055). (*S*) Average quantal content (EPSP/mEPSP) in *w^1118^* and *neto^109^* upon PhTX treatment normalized to HL3-treated controls. (*w^1118^*, *n* = 6; 213.0 ± 19.166; *P* = 0.003; *neto^109^*, *n* = 7; 93.76 ± 14.843; *P* = 0.70). Scale bars: (*A*, *Left*) 1 µm; (*A*, *Right*) 200 nm; (*B*, *Left*): 1 µm; (*B*, *Right*) 200 nm; (*E*) 1 µm; (*F*) 1 µm; (*J*) 500 nm; (*J, i*) 200 nm; (*K*) 500 nm; (*K, i*) 200 nm; (*O*) EPSP: horizontal scale: 50 ms; vertical scale: 10 mV; mEPSP: horizontal scale: 2 s; vertical scale: 2 mV. a.u., arbitrary unit; Dist, distance; int, intensity; norm, normalized; rel, relative; ns, not significant; ** *P* ≤ 0.01; *** *P* ≤ 0.001; **** *P* ≤ 0.0001.

Based on our findings that 1) Neto-β aligns with GluRs and Brp, 2) Neto stabilizes GluRs inside and outside the rings, and 3) that both GluRs and Brp undergo rapid changes during homeostatic plasticity, we hypothesized that Neto-β is modulated during homeostatic plasticity. Whereas PhTX application for 5 min did not result in apparent changes in Neto-β fluorescence intensity in wild type (*SI Appendix*, Fig. S12 *A*, *B* and *D*), PhTX application for 15 min resulted in a significant increase in the mean Neto-β fluorescence intensity at confocal resolution compared with that of saline-treated controls (Fig. 5 *A*–*C*). (HL3, 5 min: *n* = 3,278; PhTX, 5 min: *n* = 772; *P <* 0.0001; HL3, 15 min: *n* = 3,278; PhTX, 15 min: *n* = 772; *P <* 0.0001; Fig. 5 *A*–*C* and *SI Appendix*, Fig. S12 *A*, *B* and *D*). We also detected increased GluRIID intensity upon 15 min of PhTX treatment, similar to GluRIIC, providing independent evidence for GluR modulation during PHP (*SI Appendix*, Fig. S12 *E*–*G*; HL3: *n* = 737; PhTX: *n* = 507; *P* < 0.0001). Additionally, we detected a significant increase in Neto-β cluster number per ring (Fig. 5*D*), as well as an increased fraction of Neto-β–aligned GluRIID clusters (*SI Appendix*, Fig. S12 *I*–*K*) upon PhTX treatment for 15 min using gSTED imaging (Fig. 5*D*: HL3: *n* = 268; PhTX: *n* = 237; *P* < 0.0001; *SI Appendix*, Fig. S12*K*: HL3: *n* = 88; PhTX *n* = 143; *P* < 0.0001), suggesting the modulation of nanocluster abundance of the auxiliary GluR subunit Neto-β during homeostatic plasticity. By extension, these data provide independent evidence for the modulation of postsynaptic nano-organization during homeostatic plasticity.

Given the changes in Neto-β nano-organization during PHP, we investigated if *neto* is required for the modulation of transsynaptic nano-organization during homeostatic plasticity. PhTX treatment for 15 min, a manipulation that robustly increases GluRIIC abundance in wild type (Fig. 4 *A*–*F*), did not increase but rather slightly decreased mean GluRIIC fluorescence intensity in *neto^109^* mutants at confocal resolution (HL3: *n* = 2,363; PhTX: *n* = 2,497; *P <* 0.0001; Fig. 5 *E*–*I*). Brp fluorescence intensity was significantly increased by ∼10% after 15 min of PhTX application at *neto^109^* mutant synapses (HL3: *n* = 2,162; PhTX: *n* = 1,775; *P <* 0.0001; Fig. 5 *E*–*G* and *I*) but was less pronounced than in wild type (∼23%; *P <* 0.0001; Fig. 4*I*; see below). gSTED imaging revealed a slight decrease in GluRIIC and Brp cluster number per ring upon PhTX treatment (15 min) in *neto^109^* mutants (Brp: HL3: *n* = 287; PhTX: *n* = 253; GluR: HL3: *n* = 274; PhTX: *n* = 247; both: *P <* 0.0001; Fig. 5 *J*; *i*, *K, i*; and *N*), without significant changes in GluRIIC and Brp ring dimensions (HL3: *n* = 332; PhTX: *n* = 295; both: *P <* 0.0001; Fig. 5 *L* and *M*). Hence, the rapid homeostatic increase in GluR abundance, GluR cluster number, and transsynaptic nanocolumn number requires wild-type Neto levels.

Based on the observation that the modulation of transsynaptic nano-architecture during PHP is impaired in *neto^109^* mutants, we next asked if homeostatic modulation of synaptic function is affected in this genetic background. While Neto-α has been implicated in PHP presynaptically (34), it is unknown if PHP is impaired in *neto^109^* mutants with reduced Neto-α and Neto-β levels (32, 34). PhTX application for 15 min to wild-type NMJs led to a ∼50% decrease in the amplitude of spontaneous mEPSPs compared with untreated controls (HL3: *n* = 7; PhTX: *n* = 6; *P* = 0.002; Fig. 5 *O* and *Q*), indicating GluR impairment. PhTX treatment did not change action potential (AP)–evoked EPSP amplitude in wild type (HL3: *n* = 7; PhTX: *n* = 6; *P* = 0.575; Fig. 5 *O* and *R*), translating into a significant increase in quantal content (i.e., EPSP/mEPSP) compared with controls (*n* = 6; *P* = 0.003; Fig. 5*S*), suggesting increased presynaptic release, consistent with PHP (20). The *neto^109^* mutants exhibited a pronounced decrease in mEPSP amplitude in the absence of receptor perturbation (Fig. 5 *P* and *Q*), implying impaired synaptic transmission, in line with earlier work (32). To compensate for the decrease in EPSP amplitude previously reported for *neto^109^* mutants (32), the *neto^109^* recordings were conducted at elevated extracellular Ca^2+^ concentration (0.8 mM vs. 0.35 mM; Fig. 5 *P*–*S*). In contrast to wild type, PhTX incubation led to a similar decrease in mEPSP and EPSP amplitude in *neto^109^* mutants (*n* = 7; mEPSP: *P =* 0.009; EPSP: *P* = 0.0006; Fig. 5 *P*–*R*), resulting in no change in quantal content (*n* = 7; *P* = 0.69; Fig. 5*S*). These data demonstrate impaired PHP induced by pharmacological GluR inhibition in *neto^109^* mutants. As *neto^109^* mutants also display a defect in homeostatic modulation of transsynaptic nano-architecture (Fig. 5*N*), these data provide evidence that wild-type Neto levels are required for homeostatic control of synaptic nano-architecture and function.

## Discussion

In this study, we identified a stereotypic arrangement of transsynaptically aligned molecular nanocolumns that is regulated in a modular and sequential fashion during homeostatic plasticity at the *Drosophila* NMJ. Moreover, we revealed a GluR subtype–specific nano-organization and discovered that the auxiliary GluR subunit Neto is required for rapid homeostatic modulation of transsynaptic nanocolumn number and neurotransmitter release.

Previous work demonstrated that a cluster of voltage-gated Ca^2+^ channels localizes to the Brp ring center at the *Drosophila* NMJ (2). Furthermore, Unc13A, a molecule suggested as a molecular correlate of presynaptic release sites (3, 4), forms ring-like arrays in close proximity to Brp C termini and GluRs (*SI Appendix*, Fig. S4 *A*–*D*) (4). In light of these findings, our results are consistent with a model in which Ca^2+^ influx at the Brp/AZ center induces neurotransmitter release in the nanocolumn rings. Given that the neurotransmitter content released by a single synaptic vesicle does not activate all GluRs of a given PSD at the *Drosophila* NMJ (38), and that *Drosophila* GluRs have a low glutamate affinity (30), neurotransmitter release may predominantly activate GluRs that are aligned to presynaptic release sites. Some evidence suggests that synaptic transmission predominantly occurs within transsynaptic nanocolumns (8). Hence, the transsynaptic nanocolumn rings discovered here may reflect subsynaptic transmission modules that are activated by a common Ca^2+^-channel cluster. Future work is needed to relate the molecular nanocolumn topography to synaptic physiology, for example, by assessing how many GluRs are activated by neurotransmitter release from a single synaptic vesicle. In this regard, the slight offset between Unc13A and GluR rings may indicate that a given release site may not only activate a single aligned GluR cluster but also neighboring GluR clusters, consistent with physiology data (30).

GluR subunit composition and GluR location with regard to release sites are important factors determining synaptic efficacy (39). At the *Drosophila* NMJ, the ratio of slowly and rapidly desensitizing GluRIIA- and GluRIIB-containing receptors is a key regulator of quantal size (30). We revealed that transsynaptic nanocolumns harbor a mix of GluRIIA- and GluRIIB-containing receptors, and that ambient receptors, which represent almost half of the GluRs within a PSD, mainly incorporate the GluRIIB subunit. The persistence of transsynaptic nanocolumn rings in *GluRIIA* and *GluRIIB* mutants implies that neither of these subunits alone is sufficient for ring formation or transsynaptic alignment. Previous work revealed no defects in spontaneous or AP-evoked synaptic transmission upon *GluRIIA* overexpression (30) or after *GluRIIB* loss (25). Thus, two genetic manipulations that mainly decrease ambient receptor abundance, but not receptors inside the nanocolumn ring, do not induce a corresponding decrease in synaptic transmission. This indicates that synaptic transmission is largely confined to transsynaptic nanocolumn rings and/or that synaptic transmission outside the rings is dominated by rapidly desensitizing GluRIIB-containing receptors. Moreover, our observation of increased mEPSP amplitudes in *GluRIIB^SP5^* mutants suggests that GluRIIB-containing receptors surrounding the nanocolumns have the potential to negatively regulate synaptic transmission by replacing GluRIIA-containing receptors within the nanocolumns.

A variety of auxiliary subunits control GluR assembly, trafficking, and function (40). The auxiliary GluR subunit Neto has been implicated in GluR clustering at the *Drosophila* NMJ (32). We uncovered modular ring arrays of Neto-β that transsynaptically align with Brp C termini, suggesting that this auxiliary GluR subunit is a postsynaptic element of transsynaptic nanocolumn rings. The persistence of transsynaptic nanocolumn rings in hypomorphic *neto^109^* mutants suggests that *neto* is not crucial for ring formation or transsynaptic alignment, or that the remaining Neto was sufficient for transsynaptic nanocolumn ring formation. In contrast to *neto^109^* mutants, in which both Neto-α and Neto-β levels are reduced (32), loss of Neto*-*α does not decrease GluR levels or mEPSP amplitude (34), suggesting that this Neto isoform either does not stabilize GluRs at the *Drosophila* NMJ or that there is a compensation by Neto-β. While reduced levels of ambient receptors do not impair synaptic transmission in case of *GluRIIA* overexpression (30) or in *GluRIIB^SP5^* mutants (*SI Appendix*, Fig. S7 *G* and *H*), the decreased GluR abundance within the rings of *neto^109^* mutants correlates with a decrease in spontaneous and AP-evoked synaptic transmission (Fig. 5 *Q* and *R*) (32), again implying that synaptic transmission predominantly occurs within the rings.

GluR impairment at the *Drosophila* NMJ induces a homeostatic increase in release (20, 37), and there is evidence for the modulation of presynaptic nano-architecture during this form of homeostatic plasticity (21, 22). A previous study reported increased GluR levels upon sustained pharmacological GluR inhibition for several days (16). We here demonstrate GluR modulation within 5 min after pharmacological GluR impairment that precedes the modulation of Brp, as well as Neto-β. Although we cannot exclude that other molecules are modulated prior to GluRs, or that we could not resolve small changes in Brp or Neto-β after PhTX treatment for 5 min, our data imply that GluR modulation precedes Neto-β and presynaptic regulation during homeostatic plasticity. Furthermore, GluR and Brp fluorescence intensity changes detected with confocal microscopy preceded the increase in GluR and Brp cluster numbers at STED resolution. This could either indicate that small nanostructural changes could not be detected with STED microscopy or that the modulation of transsynaptic nano-architecture lags behind the regulation of GluR and Brp levels or distribution. Similar to the data obtained with confocal microscopy, the increase in GluR cluster number preceded Brp cluster regulation upon GluR perturbation, again indicative of a temporal sequence of transsynaptic changes during PHP. Interestingly, while GluR, but not Brp cluster number increased 15 min after PhTX treatment, we noted a larger fraction of transsynaptically aligned Brp clusters. This suggests that transsynaptic nanocolumn formation likely precedes Brp cluster formation. The temporal sequence of GluR and Brp regulation may also explain the existence of GluR clusters within the ring that are not opposed by Brp. Together, these findings are consistent with a model of coordinated, transsynaptic, and modular structural plasticity during PHP that results in the addition of transsynaptic nanocolumns to the ring.

We did not observe apparent changes in GluR fluorescence intensity, GluR cluster number, or homeostatic potentiation of release upon pharmacological GluR perturbation in hypomorphic *neto^109^* mutants. This shows that wild-type Neto levels are required for homeostatic control of GluRs and presynaptic release. GluR inhibition also led to a slight but significant increase in Brp fluorescence intensity in *neto^109^* mutants, which was less pronounced than in wild type. The defect in PHP seen in *neto^109^* mutants could thus arise from impaired GluR and/or Brp regulation. Although our genetic data establish a causal relationship between the homeostatic regulation of transsynaptic nanocolumns and presynaptic release, future work is required to scrutinize the relationship between transsynaptic nano-architecture and synaptic transmission, and to dissect the molecular mechanisms controlling transsynaptic nano-architecture and its homeostatic regulation. In this regard, it will be exciting to explore which molecules are involved in transsynaptic alignment and ring formation. Synaptic cell-adhesion molecules, such as neurexins and neuroligins, represent obvious candidates.

## Materials and Methods

### Fly Husbandry, Stocks, and Handling.

All experiments involving genetically modified organisms were approved by the responsible authorities (Department of Molecular Life Sciences, University Zurich authorization A120910-4). *D. melanogaster* strains were reared under standard laboratory conditions and raised at 25 °C on standard food. Male and female third-instar larvae of the following genotypes were used: *w^1118^*, *BG57-Gal4* (a gift from Jan Pielage, Technical University Kaiserslautern), *UAS-GluRIIA* (30), *GluRIIB^SP5^* (this study), *GluRIIA^SP16^* (a gift from Graeme Davis, University of California, San Francisco), *dSol-1* (a gift from author D.D.), and *neto^109^* (a gift from Mihaela Serpe, National Institutes of Health). *GluRIIB^SP5^* mutants were generated using a CRISPR/Cas9 genome-editing strategy as previously described (41). One single guide RNA (sgRNA) line that targeted the sixth exon of the *GluRIIB* locus (sgRNA: 5′-CATTGATGGATTCTACTCCCGGG-3′) was cloned into the pU6 vector. This construct was sent to BestGene Inc. for targeted insertion into the VK18 attP site on the second chromosome. sgRNA flies were crossed to a *vas-Cas9* line on the second chromosome to induce active germline CRISPR mutagenesis, and 20 independent lines were screened by PCR for mutations. This identified eight independent insertion and deletion mutations that shifted the open reading frame. *GluRIIB^SP5^* led to an early STOP codon at the 276th amino acid (T276STOP) and was kept for additional analysis.

### Immunostaining.

*Drosophila* larvae were dissected and processed similar as described previously (36). In brief, wandering third-instar larvae were dissected in HL3 saline (in mM: 70 NaCl, 5 KCl, 10 MgCl_2_, 10 NaHCO_3_, 115 sucrose, 5 trehalose, 5 Hepes, 0.3 CaCl_2_). After dissection, preparations were washed with HL3 saline and fixed with ethanol (100% ethanol [EtOH]; Merck kGaA, 64–17-5) for 15 min on ice. For triple staining in *SI Appendix*, Fig. S4, samples were fixed with methanol (Merck kGaA, 67–56-1) for 7 min at room temperature. Thereafter, preparations were quickly rinsed three times with phosphate-buffered saline (PBS) and then thoroughly washed with PBS containing 0.1% Triton X-100 (5 × 10 min). For pharmacological GluR blockade (Figs. 4 and 5 and *SI Appendix*, Figs. S11 and S12), larvae were either incubated with HL3 (control) or the GluR antagonist PhTX (20 µM; Santa Cruz Biotechnology, SC-255421) for 5, 15, or 30 min at room temperature before applying EtOH. After washing with PBS/Triton X-100, preparations were blocked with 3% normal goat serum in PBS containing 0.1% Triton X-100 for 1.5 to 2 h. Incubation with primary antibodies was done at 4 °C on a rotating platform overnight. The following primary antibodies and dilutions were used: anti-Brp [mouse, nc82 (13); 1:100], anti-GluRIIC (rabbit, 1:100, provided by Jan Pielage, Technical University Kaiserslautern) for *neto^109^* stainings ( Figs. 3 and 5 and *SI Appendix*, Fig. S9), anti-GluRIIC was used at a dilution of 1:500; anti-GluRIIA (mouse, 1:1,000; Developmental Studies Hybridoma Bank), anti-GluRIIB (rabbit, 1:2,000; D.D. laboratory), anti-Neto-β (rabbit, 1:500; D.D. laboratory), anti-Unc13A (rabbit, 1:300; provided by Stephan Sigrist, Freie Universität Berlin), anti-GluRIID (guinea pig, 1:1,000; D.D. laboratory). The following secondary antibodies were applied for 2 h at room temperature on a rotating platform: Atto 594 (anti-mouse, 1:100; Sigma-Aldrich, 76085), Abberior STAR 635 P (anti-rabbit, 1:100; Abberior, 53399), Abberior STAR 635 P (anti-guinea pig, 1:200; Abberior ST635P-1006). For triple staining in *SI Appendix*, Fig. S4, Atto 490 LS (anti-guinea pig) was used at 1:200. For *neto^109^* stainings (Figs. 3 and 5 and *SI Appendix*, Fig. S9), Abberior STAR 635 P (anti-rabbit) was used at a dilution of 1:250. Preparations were mounted onto slides with ProLong Gold (Life Technologies, P36930). Experimental groups of a given experiment were processed in parallel in the same tube.

### Image Acquisition and Processing.

Confocal and gSTED microscopy were performed with an inverse Leica TCS SP8 STED 3X microscope (Leica Microsystems) at the University of Zurich Center for Microscopy and Image Analysis. For excitation, we used a flexible white-light laser with an output range of 470 to 670 nm in combination with a 775-nm STED depletion laser. Excitation light (488 nm, 580 nm, or 640 nm) was focused onto the specimen using a ×100 objective (HC PL APO 1.40 NA Oil STED White; Leica Microsystems) with immersion oil conforming to ISO 8036 with a diffraction index of *n* = 1.5180 (Leica Microsystems). Emitted light was detected with two HyD detectors in photon-counting mode (Leica Microsystems). For STED imaging, we used time-gated single photon detection [empirical adjustment within a fluorescence lifetime interval from 0.7 to 6.0 ns (42)]. Pixel size was 10 × 10 nm or 20 × 20 nm, and z-stacks were acquired with a step size of 120 or 130 nm. Line accumulation was set to 1 and 6 for confocal and STED imaging, respectively. Images were acquired with LAS X software (Leica Application Suite X, version 2.0; Leica Microsystems). Experimental groups were imaged side by side with identical settings. In the time-course experiments (Fig. 4*I* and *SI Appendix*, Fig. S11 *A*–*C*), data were obtained with identical settings for a given time point, but with different settings between time points to prevent fluorescence intensity saturation.

Images were processed and deconvolved with Huygens Professional (Huygens compute engine 17.04, Scientific Volume Imaging B.V.). In brief, the automatic background detection tool (radius = 0.7 µm), and the auto stabilize feature were used to correct for background and lateral drift. Images were deconvolved using the Good’s roughness maximum likelihood algorithm with default parameter settings (*n* = 10 maximum iterations; signal to noise ratio: 7 for STED; quality threshold: 0.003). ImageJ (version 1.51n; National Institutes of Health) was used for maximum-intensity z-projections.

### Electrophysiology.

Wandering third-instar larvae were dissected in HL3 solution (5 mM KCl, 70 mM NaCl, 10 mM Na-Hepes, 5 mM Hepes, 5 mM trehalose, 115 mM sucrose, 10 mM MgCl_2_) with 0.35 or 0.8 mM CaCl_2_ for sharp-electrode membrane-voltage recordings. The internal organs, including the CNS and the ventral nerve cord, were carefully removed from the body wall with intact muscle fibers and innervating motor nerves. Sharp-electrode recordings were performed on muscle 6 of segments 3 and 4 with sharp borosilicate glass electrodes (resistance, 10 to 25 MΩ) using an Axoclamp 900A amplifier (Molecular Devices). For individual NMJs, mEPSPs were recorded prior to EPSPs induced by stimulating the respective hemisegmental nerve with single APs (3-ms stimulus duration, 0.3 Hz). A total of 30 EPSPs were recorded to obtain the mean EPSP amplitude for each cell. mEPSPs were analyzed from a 5-min recording.

Semi-intact larvae (dorsally dissected, nonstretched, with internal organs, CNS and ventral nerve cord intact) (20) were incubated with the GluR antagonist PhTX-433 (20 µM; catalog sc-255421, Santa Cruz Biotechnology) for ∼15 min. This was followed by HL3 washes, removal of internal organs, CNS, and ventral nerve cord to obtain a fully dissected preparation for electrophysiological recordings (20).

Electrophysiology data were acquired with Clampex (Molecular Devices) and analyzed using routines written with scientific python libraries, including numpy, scipy, IPython, and neo (43). mEPSPs were detected using an implementation of a template-matching algorithm (44, 45). Quantal content was calculated as the ratio between the mean EPSP amplitude and the mean mEPSP amplitude for each cell.

### Quantification and Statistical Analysis.

Data were analyzed using Prism (GraphPad Software) and IgorPro, version 6.37 (WaveMetrics Inc.). Data in the text are generally reported as mean ± SEM. Data distributions were tested with the D'Agostino and Pearson normality test. Line-profile intensities are represented as mean ± SEM, and line-profile intensities in Figs. 1*C*; 2 *D*, *H*, *I*, *M*, and *N*; 3 *D*, *H*, and *I*; 4 *L* and *M*; 5 *L* and *M*; and *SI Appendix*, Fig. S8 were compared using a Kruskal-Wallis test and a Dunn's post hoc multiple comparison test. Cluster counts in Figs. 2 *E*, *J*, and *O*; 3 *E* and *J*; 4*N*; 5*N*; and *SI Appendix*, Figs. S5*I*, S8*C*, and S10*C* are shown as box-and-whisker plots (minimum, first quartile, median, third quartile, and maximum). Cluster counts, unless otherwise stated, were analyzed using an ordinary one-way ANOVA with a Tukey’s post hoc multiple comparison test (if data were normally distributed) or with a Kruskal-Wallis test with Dunn’s post hoc multiple comparison test (if data were nonnormally distributed). Exceptions were: unpaired Student’s *t* test with Welch’s correction in Figs. 1 *E* and 2*E*, Mann-Whitney test (Fig. 3*E*), ordinary one-way ANOVA with Bonferroni’s post hoc multiple comparison test (Fig. 3*J*), and a one sample *t* test (*SI Appendix*, Fig. S11). Cumulative frequency distributions were analyzed with a Kolmogorov-Smirnov test (Figs. 4 *G* and *H*, and 5 *G* and *H*). Brp-fluorescence intensity data in *neto^109^* were compared with *w^1118^* using a Mann-Whitney test. Sample sizes and *P* values are reported in the text, figure legends, and in the supplementary tables. Unless otherwise noted, *n* refers to the number of puncta (confocal), or detected rings, or clusters per ring (STED). Unless otherwise noted, data are based on *N* ≥ 4 NMJs. We ran a linear mixed model (Multicomp package in R, version 3.5.3) to test if the statistical differences of the recorded parameters depended on our sample size definition by considering NMJ number (*N*) and AZ number (*n*) as random effects, and treatment condition (HL3 vs. PhTX) as the fixed effect for one of our largest data sets (ring diameter and cluster number, 30-min HL3 vs. PhTX; Fig. 4). The results of the linear mixed model suggest that the statistical differences observed in this data set are due to the fixed effect (treatment) rather than the random effect (*N* vs. *n*), suggesting that our conclusions are independent of our sample size definition.

## Supplementary Material

Supplementary File

## Data Availability

All data of this study are included in the article and/or the *SI Appendix*. All materials and code will be provided upon reasonable request.
