# Supplementary material for: Rapid homeostatic modulation of transsynaptic nanocolumn rings
Source: Proc Natl Acad Sci U S A. 2022 Nov 2;119(45):e2119044119. doi: 10.1073/pnas.2119044119 (PMC9659372; doi:10.1073/pnas.2119044119)
Supplement: Supplementary File [file pnas.2119044119.sapp.pdf]

Supplementary Materials for

Rapid homeostatic modulation of transsynaptic nanocolumn rings

Paola Muttathukunnel[1,2], Patrick Frei[1], Sarah Perry[3], Dion Dickman[3] and Martin Müller[1,2,*]

[1]Department of Molecular Life Sciences, University of Zurich, Winterthurerstrasse 190, 8057 Zurich, Switzerland

[2]Neuroscience Center Zurich, University of Zurich/ETH Zurich, Zurich, 8057 Zurich, Switzerland

*Corresponding author: Martin Müller

Email: Martin.Mueller@mls.uzh.ch

This PDF file includes:

Supplementary Material and Methods
Supplementary Figures S1 to S12
Supplementary Figure Legends S1 to S12

Supplementary Material and Methods:

Image Analysis

Fluorescence intensity and area: Fluorescence intensity and area were analyzed from max. intensity z-projection confocal data using Fiji/ImageJ (version 1.51n, National Institutes of Health, USA). To create a binary mask, max. projection intensity data were background corrected (rolling ball, radius 1 = µm) and filtered (3 x 3 median). Thereafter, a threshold was set to 15 – 30% of the maximum fluorescence intensity of a pixel located within the NMJ for the Brp- and GluR channel. The resulting binary mask was then projected onto the respective background-corrected, unfiltered max. projection data and the intensity and area of individual particles were read out.

Ring detection: Ring detection was implemented in C++ using the open source (CeCILL license) "CImg" library (cimg.eu/) for image loading/storage. In brief, the entire deconvolved max. z-projection was scanned pixel-by-pixel in eight directions with regard to a given center pixel with a filter (Figure S2A) to assign each pixel with a ring-center probability (Figure S2B). The filter accumulates fluorescence intensity relative to its center in eight directions (two horizontal, two vertical and four diagonal) within a distance of 40 – 120 nm from the center pixel (Figure S2A). For each pixel, the accumulated values for each direction are added to a score, which is compared to two thresholds. First, the number of directions with filter values larger than the "high threshold" (t_h) is counted. t_h was defined as the maximum score multiplied by an empirically-derived constant of 0.05 (see also below, "*Line profiles*"). Second, the number of directions with filter values larger than the low threshold (t_l, defined as the maximum score multiplied by a constant of 0.025) is counted. Based on the eight directions, both values are within a range from zero to eight. A ring-center probability larger than zero is assigned to a pixel if both values are larger than six. The threshold of six is based on calibration experiments in which we systematically changed the threshold and visually inspected the results. Pixels with the highest ring-center probability are first assigned as "ring centers". If two pixels with ring-center probabilities greater than zero are closer than 10 pixels (200 nm) distance, only the pixel with the higher probability is assigned as a ring center. Note that the algorithm considers local fluorescence gradients rather than a threshold.

Line profiles: Line profiles with a length of 600 nm were either independently anchored in Brp- and GluR-ring centers (Figure S2D), or in Brp-ring centers alone (Figure S2D, Figure 1 – 5 and Figure S4-10). For each line profile, we read out the Brp- and GluR fluorescence intensity along a line that was rotated by 360 degrees in single degree steps, and centered at the respective ring center. Normalized line profiles were obtained by dividing the profile of each ring by its fluorescence intensity maximum. Based on different line-profile shapes, the peaks of the average line profiles are smaller than one. Average line profiles are based on all rings detected in a given experimental group. Line profiles with a fluorescence maximum within a diameter of <100 nm with respect to the ring center were defined as "not ring-like" (Figure S2F). The ring detection algorithm was adjusted such that the fraction of "not ring-like" line profiles was close to zero in the Brp channel. Ring diameter was quantified as the distance between the local maxima of the line profiles.

Local maximum detection: For the detection of local maxima, i.e. 'nano-clusters' within a ring (Figure 1D', 1E, Figure S3A, S3B), two criteria were applied: The first criterion assigns high cluster-center probabilities to pixels with high fluorescence intensity relative to neighboring pixels within a distance of one to three pixels (Figure S3A). The second criterion is in place to evaluate adjacent pixels and to exclude 'isolated' pixels with high fluorescence intensity. Pixels with fluorescence intensities above a threshold of 20 a.u. (8-bit) get initialized with the maximum cluster-center probability, while pixels with intensities below this threshold get assigned with a cluster-center probability of zero. Then, the fluorescence intensity ratios between nearby pixels around a potential cluster center are analyzed. Nearby pixels in a range from one to three are considered according to the following scheme (Figure S3A): Depending on the distance to the center, these nearby pixels are assigned with different intervals (distance of one pixel: 0.60 - 0.97; distance of two pixels: 0.32 - 0.98; distance of three pixels: 0.01 - 0.99). If the calculated intensity ratio of a nearby pixel to the center pixel is within the specific interval, the cluster- center probability of the center pixel is not decreased. If the calculated intensity ratio is outside the interval, the probability of the center pixel is decreased by the distance of the ratio to the nearest point of the interval and multiplied by a factor of 50. For all pixels with a cluster-center likelihood greater than a threshold of 20 a.u., a second criterion is applied in two steps as follows: First, if any pixel within a distance of one or two pixels in x or y direction shows a higher intensity than the center pixel, the respective center pixel is no longer considered as a potential cluster center. If e.g. two pixels next to each other with a symmetrical intensity decrease in the nearby region have a cluster center probability greater than zero assigned from the first criteria, only the brighter pixel keeps its cluster center likelihood, while the other pixel gets assigned with a likelihood of zero. Second, if more than three of the eight pixels

surrounding the center pixel have an intensity below 10 a.u., the center pixel gets assigned with a cluster center probability of zero. With this rule, isolated bright pixels surrounded by very low-intensity pixels are excluded as cluster centers. On our images, these cases most likely represent noise, as they are evenly distributed over the whole image. Finally, pixels with the highest cluster-center probability get assigned as cluster centers first. Subsequently, pixels with lower cluster-center probabilities get assigned as cluster centers. If two pixels with cluster-center probabilities greater than zero are closer than three pixels distance, only the pixel with the higher probability gets assigned as a cluster center. This fluorescence intensity gradient-based approach allows detecting local maxima in heterogeneous fluorescence intensity landscapes (Figure S2A).

Transsynaptic cluster alignment (Figure 4O, Figure S4E-F, S6I, S9G-H and S12K) was manually scored by counting the number of unaligned clusters per ring, as defined by no fluorescence overlap between the channels, and normalizing the number of the remaining aligned clusters to the total cluster number per ring (Figure S4E).

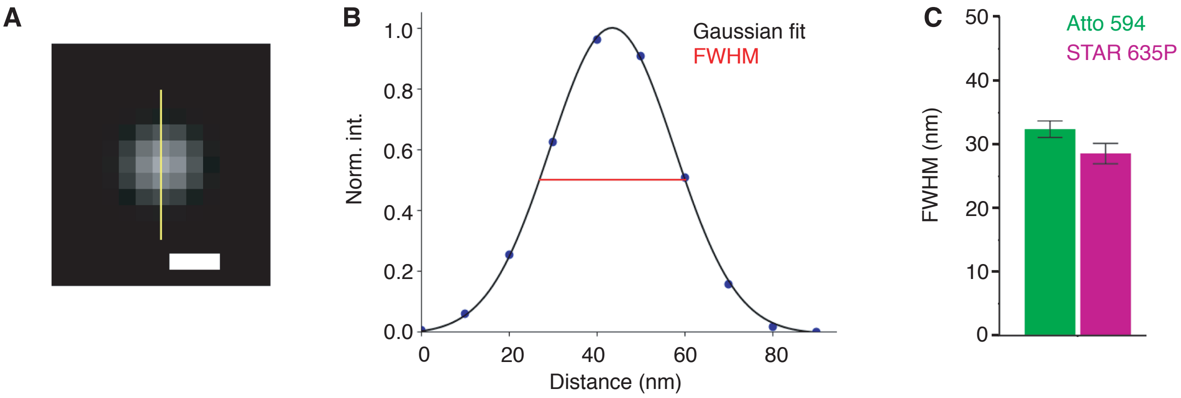
A
B
1.0
0.8
0.6
0.4
0.2
0.0
Norm. int.
0
20
40
60
80
Distance (nm)
Gaussian fit
FWHM
C
50
40
30
20
10
0
FWHM (nm)
Atto 594
STAR 635P

Fig. S1.

Spatial resolution. (A) Representative image of a dual-color fluorescent bead (ATTO 594, STAR 635P; diameter, *d* = 23nm; GattaQuant) excited with a white-light laser (594 nm) and time-gated STED depletion (775 nm; 2D STED) after Huygen's deconvolution (see SI). The line indicates the location of the intensity profile shown in (*B*). **(B)** Peak-normalized fluorescence intensities (*blue*) and Gaussian fit (*black*) as a function of lateral distance of the data highlighted by the line in *(A)*. The red line represents the full width at half maximum (FWHM). **(C)** Average FWHM values for ATTO 594, STAR 635P (±SEM, *n* = 15 beads). Note the effective lateral resolution of ~30 nm. Scale bar: (*A*) 50 nm.

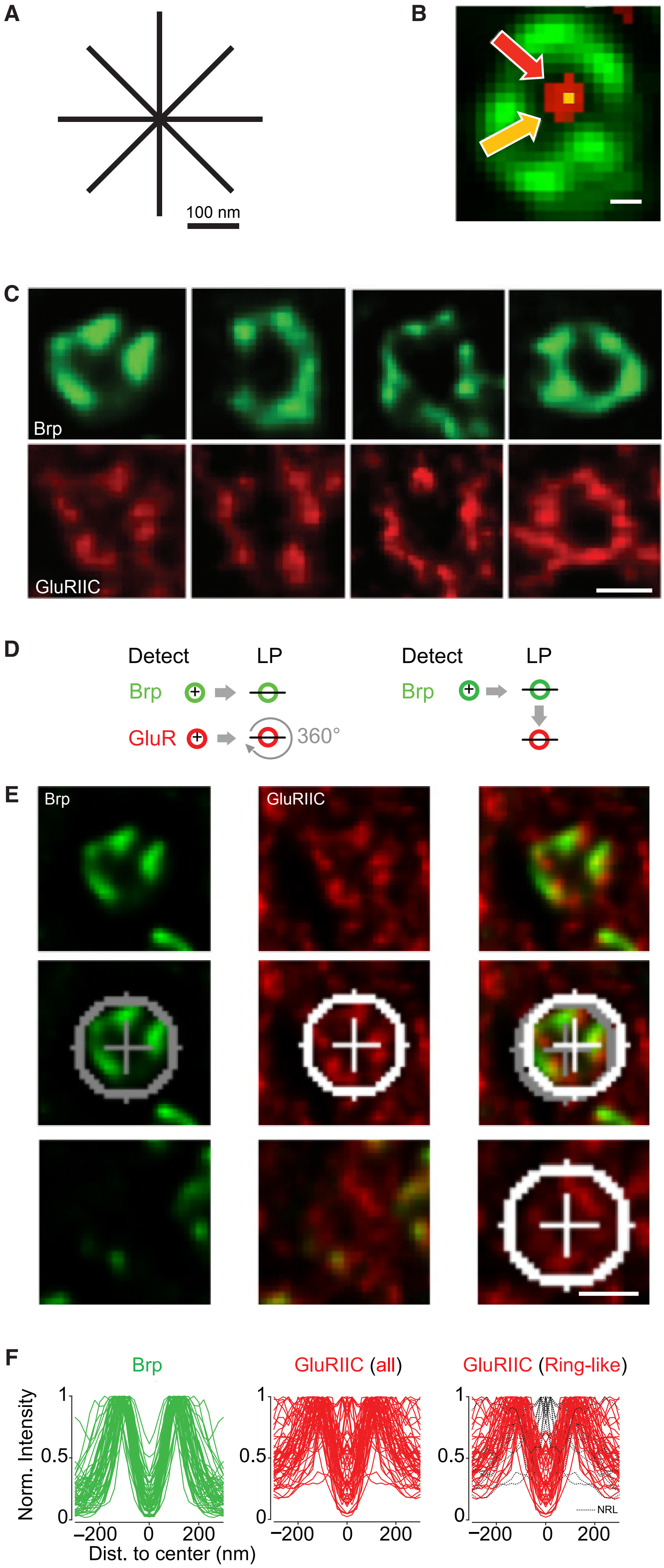
A
100 nm
B
C
Brp
GluRIIC
D
Detect
LP
Brp
GluR
360°
Detect
LP
Brp
E
Brp
GluRIIC
F
Brp
GluRIIC (all)
GluRIIC (Ring-like)
Norm. Intensity
1
0.5
0
−200
0
200
Dist. to center (nm)
NRL

Fig. S2.

Ring detection and line profile analysis. (A) Schematic of the filter used for ring detection. Fluorescence intensity gradients are read out in 8 directions with respect to a center pixel. For details see section *'Image analysis'/'Ring detection'* (see SI). **(B)** Example of ring-center probability estimation for a wild-type Brp ring. The yellow pixel denotes the estimated ring center. Red pixels show pixels with lower ring-center probabilities surrounding the center. **(C)** Examples of opposed wild-type Brp- and GluRIIC rings. (**D**) Schematic of the line profile ("LP") analysis. LPs were read out after either independent Brp- and GluR-ring detection (*left*) or after Brp-ring detection alone (*right*). The crosses symbolize ring detection. **(E)** Example of detected wild-type Brp- and GluRIIC rings. The upper two rows show examples in which Brp- and GluRIIC-rings were independently detected in close proximity. An example of a GluRIIC ring that was detected without an apparent opposing Brp ring is shown in the bottom row. (**F**) Representative Brp- and GluRIIC-line profiles of a w^{1118} NMJ, with all GluRIIC-line profiles detected opposite to the Brp-ring centers ('GluRIIC (all)', *midde*), and GluRIIC-line profiles without a local maximum at the center and/or a diameter <100 nm ('GluRIIC (Ring like)'; non-ring-like ('NRL') line profiles are shown as black dashed lines, *right*). Data represent individual line profiles (n = 55 rings) obtained from a w^{1118} NMJ. Scale bars: (B) 100 nm; (C, E) 200 nm.

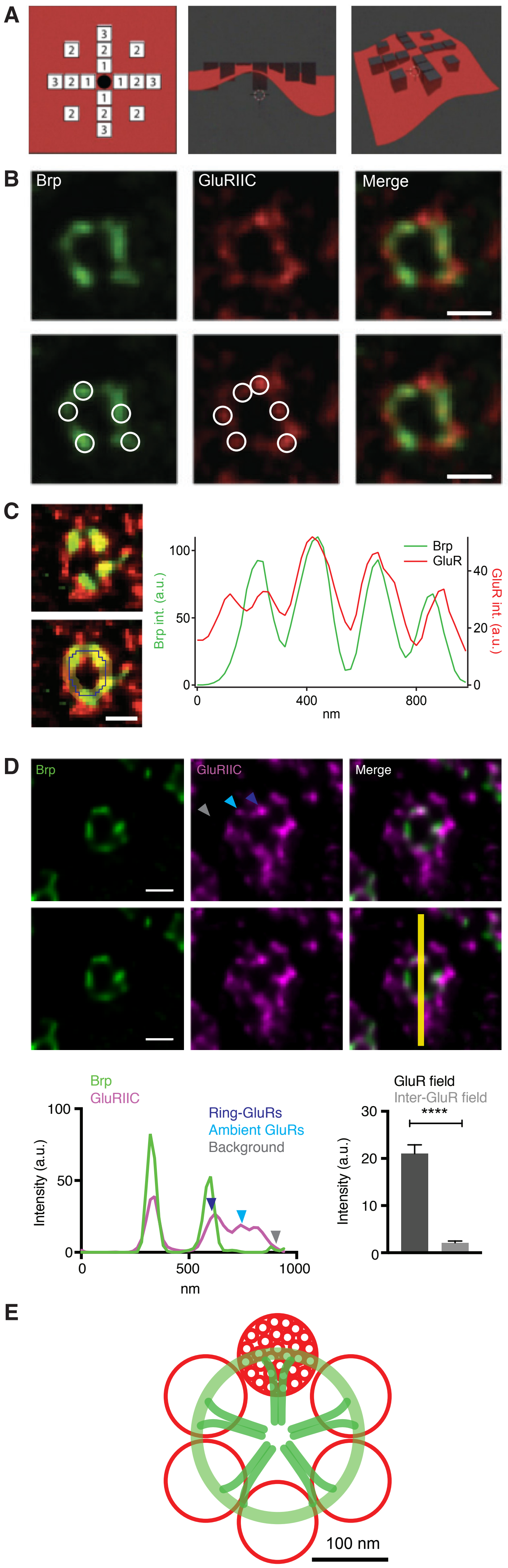
A
3
2
1
B
Brp
GluRIIC
Merge
C
Brp
GluR
100
50
0
Brp int. (a.u.)
40
20
GluR int. (a.u.)
0
400
800
nm
D
Brp
GluRIIC
Merge
Ring-GluRs
Ambient GluRs
Background
Intensity (a.u.)
500
1000
GluR field
Inter-GluR field
****
30
10
E
100 nm

Fig. S3.

Local maximum detection. (A) Schematic of pixel the filter used for local maximum ('cluster') detection. (*Left*) Top view, white squares represent filter-element positions. Numbers in squares denote filter-element ranges. Side and oblique view are shown in the *middle* and on the *right*. The red surface represents fluorescence intensities (surface is shown as a continuum for illustration purposes). For details see *'Image analysis'/'Local maximum detection'* (see SI). **(B)** gSTED maximal projection of a representative Brp- and GluRIIC ring with local maxima detected by the algorithm (white circles). **(C)** (*Left*) Representative example of a Brp ring (green) and a corresponding GluRIIC ring (red). The blue line indicates the location of the intensity profile along the Brp ring. (*Right*) Mean intensity profile (average of three pixels) of the Brp (green) and GluRIIC (red) channel of the example shown on the left. Note the overlap of most local maxima between the two channels, indicating transsynaptic alignment of Brp- and GluRIIC clusters, as well as an unaligned GluR cluster. **(D)** (*Top*) Example of Brp (green) and opposed GluRIIC (magenta) rings. GluRs within the ring, ambient GluRs outside the ring, and background fluorescence are indicated by dark blue, light blue and gray arrowheads, respectively. The yellow line demarks the location of the intensity profile (*bottom, left*; same color code for arrowheads as above). (*Bottom, right*) Mean anti-GluRIIC intensity within GluR fields (dark gray) and between GluR fields (light gray) quantified from gSTED max. projections (n = 20; P < 0.0001). Note that the background GluRIIC fluorescence in between the GluR fields is significantly lower than for ambient GluRs and ring GluRs. **(E)** Schematic representation of a back-of-the-envelope estimation of the maximal GluR number per cluster. Based on the average diameter of Brp- and GluR rings (~200 nm; green circle) and the average GluR-cluster number (~6), each GluR cluster (large red circles) has a maximal diameter of ~100 nm. Assuming a GluR diameter of ~15 nm (note that real GluRs are elliptic with a major axis of ~15 nm; e.g. (37), a given cluster can harbor up to ~32 GluRs (small red circles; (41). We consider this as an overestimation, as there are additional proteins, such as auxiliary GluR subunits. Scale bars (B - D) 200 nm; (E) ~100nm.

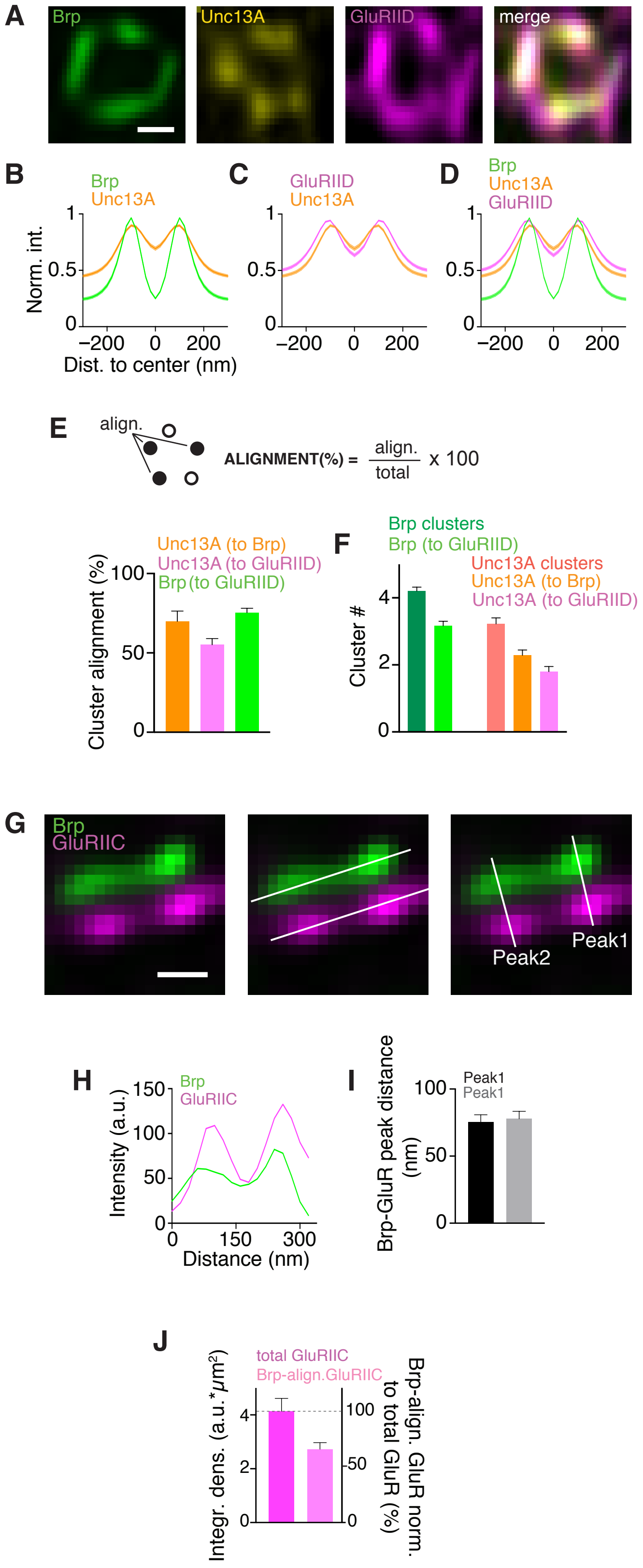
A
Brp
Unc13A
GluRIID
merge
B
Brp
Unc13A
Norm. int.
Dist. to center (nm)
C
GluRIID
Unc13A
D
Brp
Unc13A
GluRIID
E
align.
ALIGNMENT(%) = align. / total x 100
Unc13A (to Brp)
Unc13A (to GluRIID)
Brp (to GluRIID)
Cluster alignment (%)
F
Brp clusters
Brp (to GluRIID)
Unc13A clusters
Unc13A (to Brp)
Unc13A (to GluRIID)
Cluster #
G
Brp
GluRIIC
Peak1
Peak2
H
Brp
GluRIIC
Intensity (a.u.)
Distance (nm)
I
Peak1
Peak1
Brp-GluR peak distance (nm)
J
total GluRIIC
Brp-align.GluRIIC
Integr. dens. (a.u.*μm²)
Brp-align. GluR norm. to total GluR (%)

Fig. S4. Unc13A-Brp-GluRIID triple staining and vertical synapses. (A) Example of Brp- (green), Unc13A- (yellow) and GluRIID (magenta) rings observed at w^{1118} synapses at STED resolution. (**B**-**D**) Corresponding Brp- and Unc13A- (**B**), GluRIID- and Unc13A- (**C**) and Brp, Unc13A and GluRIID (**D**) line profiles. GluRIID and Unc13A line profiles were read out after Brp ring detection (*n* = 174; the shaded area represents s.e.m.). (**E**) (*Top*) Schematic of quantification of aligned clusters: aligned clusters (black; in example: 3) were normalized to total clusters (all clusters; in example: 5) per ring. (*Bottom*) Percentage (see **E**) of Unc13A clusters aligned to Brp (orange), Unc13A clusters aligned to GluRIID (pink) and Brp clusters aligned to GluRIID (green) (*n* = 49; Unc13A (to Brp): 69.84% ± 6.503; Unc13A (to GluRIID): 55.10% ± 3.904; Brp (to GluRIID): 75.34% ± 2.781). (**F**) Number of Brp (dark green) and Unc13A clusters (light red) and quantification (see **E**) of Brp clusters aligned to GluRIID (green), Unc13A clusters aligned to Brp (orange) and Unc13A clusters aligned to GluRIID (pink) (cluster number: *n* = 49; Brp: 4.204 ± 0.117; Brp (to GluRIID): 3.163 ± 0.138; Unc13A: 3.224 ± 0.178; Unc13A (to Brp): 2.286 ± 0.160; Unc13A (to GluRIID): 1.796 ± 0.154). (**G**) Example of a Brp-GluR synapse from an oblique perspective (vertical synapse, *left*). The horizontal white lines indicate the location of the intensity profile along the oblique Brp AZ (*middle*). The vertical lines indicate the location of the Brp-GluR peak distance across the Brp-GluR oblique synapse (*right*). (**H**) Corresponding intensity line profile based on the horizontal white lines (see **G**; example profile from one oblique ring shown in G). (**I**) Corresponding average Brp-GluR peak distance (*n* = 45; Peak1: 75.56 nm ± 5.199; peak2: 77.78 nm ± 5.586). (**J**) Integrated density (left y-axis) and of total GluR (dark pink) vs. GluR in the ring (Brp-aligned GluR, light pink) and percentage of GluR found in the ring (right y-axis) (*N* = 7; total GluRIIC: 4.134 a.u.*μm^2 ± 0.48; % = 100; Brp-align. GluRIIC: 2.729 a.u.*μm^2 ± 0.240; % = 65.91). Scale bars: (A) 100 nm; (G) 100 nm.

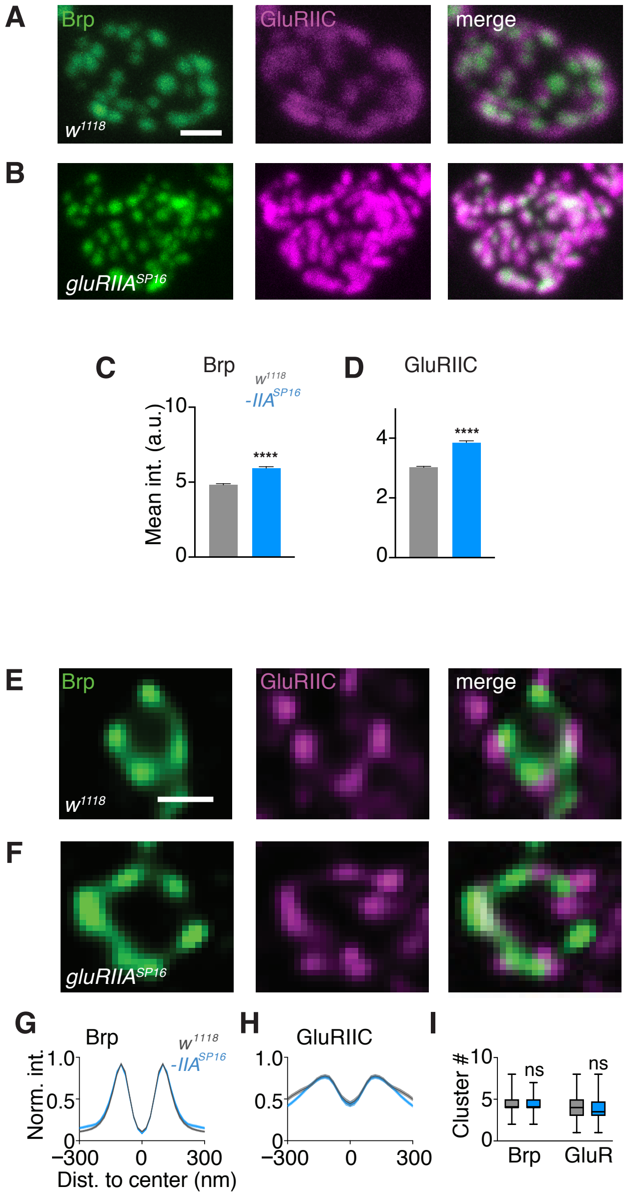

A
Brp
GluRIIC
merge
w1118
B
gluRIIASP16
C
Brp
w1118
-IIASP16
Mean int. (a.u.)
10
5
0
****
D
GluRIIC
4
2
0
****
E
Brp
GluRIIC
merge
w1118
F
gluRIIASP16
G
Brp
w1118
-IIASP16
Norm. int.
1.0
0.5
0
−300
0
300
Dist. to center (nm)
H
GluRIIC
1.0
0.5
0
−300
0
300
I
Cluster #
10
5
0
ns
ns
Brp
GluR

Fig. S5. GluRIIA-mutant. (**A**-**B**) Representative confocal images of *w*^{1118} (A) and *gluRIIA*^{SP16} (B) boutons stained with anti-Brp (green) and anti-GluRIIC (magenta). (**C**-**D**) Corresponding average mean intensity for Brp and GluRIIC in *w*^{1118} (gray) and *gluRIIA*^{SP16} (blue) (Brp: *n*- *w*^{1118} = 709; 4.813 a.u. ± 0.087; *n*-*gluRIIA*^{SP16} = 475; 5.930 a.u. ± 0.097; *P* < 0.0001; GluRIIC: *n*- *w*^{1118} = 877; 3.022 a.u. ± 0.032; *n*-*gluRIIA*^{SP16} = 595; 3.850 a.u. ± 0.062; *P* < 0.0001). (**E**-**F**) Representative Brp (green) and GluRIIC (magenta) rings in *w*^{1118} (E) and *gluRIIA*^{SP16} (F) at STED resolution. (**G**-**I**) Normalized intensity line profiles and average cluster number of Brp and GluRIIC in w^{1118} (gray) and *gluRIIA*^{SP16} (blue). (G-H: *n*- *w*^{1118} = 108; *n-gluRIIA*^{SP16} = 120 I: cluster number: Brp: *n*- *w*^{1118} = 108; 4.333 ± 0.112; *n-gluRIIA*^{SP16} = 120; 4.167 ± 0.081; *P* = 0.893; GluRIIC: *n*- *w*^{1118} = 104; 3.837 ± 0.155; *n-gluRIIA*^{SP16} = 120; 3.617 ± 0.131; *P* = 0.212). Scale bars: (A) 1 µm; (E) 200 nm.

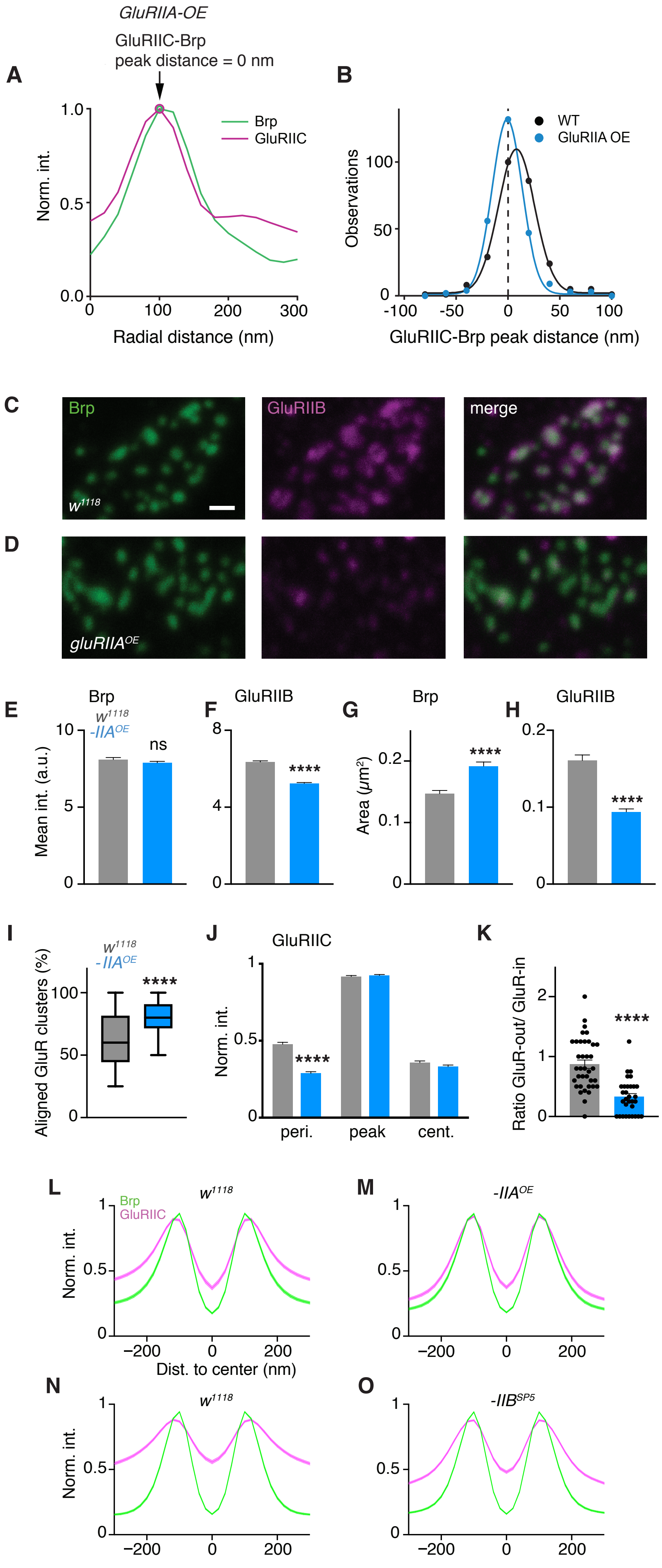

GluRIIA-OE
GluRIIC-Brp
peak distance = 0 nm
A
Brp
GluRIIC
Norm. int.
Radial distance (nm)
B
WT
GluRIIA OE
Observations
GluRIIC-Brp peak distance (nm)
C
Brp
GluRIIB
merge
w1118
D
gluRIIA^OE
E
Brp
w1118
-IIA^OE
ns
Mean int. (a.u.)
F
GluRIIB
****
G
Brp
Area (μm²)
****
H
GluRIIB
****
I
w1118
-IIA^OE
Aligned GluR clusters (%)
****
J
GluRIIC
Norm. int.
****
peri.
peak
cent.
K
Ratio GluR-out/ GluR-in
****
L
w1118
Brp
GluRIIC
Norm. int.
Dist. to center (nm)
M
-IIA^OE
N
w1118
Norm. int.
O
-IIB^SP5

Fig. S6 *GluRIIA*-overexpression (*GluRIIA-OE*). (A) Normalized fluorescence intensity line profile as a function of radial ring distance for a representative Brp ring (green) and an opposed GluRIIC ring (magenta) of wild-type (w^{1118}). The peak of the Brp- and GluRIIC line profile is marked by a green and a magenta circle, respectively. **(B)** Histograms of GluRIIC-Brp peak distance in w^{1118} (black) and after postsynaptic GluRIIA overexpression (*BG57-Gal4* > *UAS-GluRIIA*, blue). Note the improved GluR-Brp alignment upon GluRIIA-OE (w^{1118}: *n* = 266; 13 nm ± 2; *GluRIIA-OE: n* = 256; 1.3 nm ± 1). (**C**-**D**) Representative confocal boutons stained with anti-Brp (green) and anti-GluRIIC (magenta) in w^{1118} and *GluRIIA-OE*. (**E**-**F**) Average mean intensity of Brp and GluRIIB in w^{1118} (gray) and *GluRIIA-OE* (blue) (Brp: *n*-w^{1118} = 937; 8.097 a.u. ± 0.128; *n-GluRIIA-OE* = 649; 7.886 a.u. ± 0.093; *P* = 0.092; GluRIIB: *n*-w^{1118} = 1700; 6.350 a.u. ± 0.06; *n-GluRIIA-OE* = 1593; 5.240 a.u. ± 0.041; *P* < 0.0001). (**G**-**H**) Area of Brp and GluRIIB in the indicated genotypes (Brp: *n*-w^{1118} = 937; 0.147 μm^2 ± 0.005; *n-GluRIIA-OE* = 649; 0.192 μm^2 ± 0.007; *P* < 0.0001; GluRIIB: *n*-w^{1118} = 1700; 0.161 μm^2 ± 0.007; *n-GluRIIA-OE* = 1593; 0.094 μm^2 ± 0.004; *P* < 0.0001). (**I**) Percentage of GluRIIC clusters aligned to Brp in w^{1118} (gray) and *GluRIIA-OE* (blue) (*n*-w^{1118} = 61; 64.45% ± 2.757; *n-GluRIIA-OE* = 81.14% ± 1.862; *P* < 0.0001). (**J**) Normalized GluRIIC intensity at the ring periphery, peak and ring center (*n*-w^{1118} = 268; *n-GluRIIA-OE* = 257; periphery: w^{1118}*:* 0.477 ± 0.012; *GluRIIA-OE:* 0.288 ± 0.011; *P* < 0.0001; peak: w^{1118} = 0.917 ± 0.006; *GluRIIA-OE* = 0.924 ± 0.007; *P* > 0.999; center: w^{1118} = 0.357 ± 0.012; *GluRIIA-OE* = 0.332 ± 0.011; *P* > 0.999). (**K**) Ratio of GluRIIC clusters found outside - over GluRIIC clusters found at the GluR rings (*n*-w^{1118} = 37; 0.873 ± 0.070; *n-GluRIIA-OE* = 31; 0.329 ± 0.054*; P* < 0.0001). (**L**-**O**) Brp-GluRIIC channel comparison in *GluRIIA-OE* and $gluRIIB^{SP5}$ and their respective w^{1118} controls (L-M: *n*-w^{1118} = 247; *n-GluRIIA-OE* = 282; N-O: *n*-w^{1118} = 374; *n*-$GluRIIB^{SP5}$ = 573). Scale bar: (C) 1 µm.

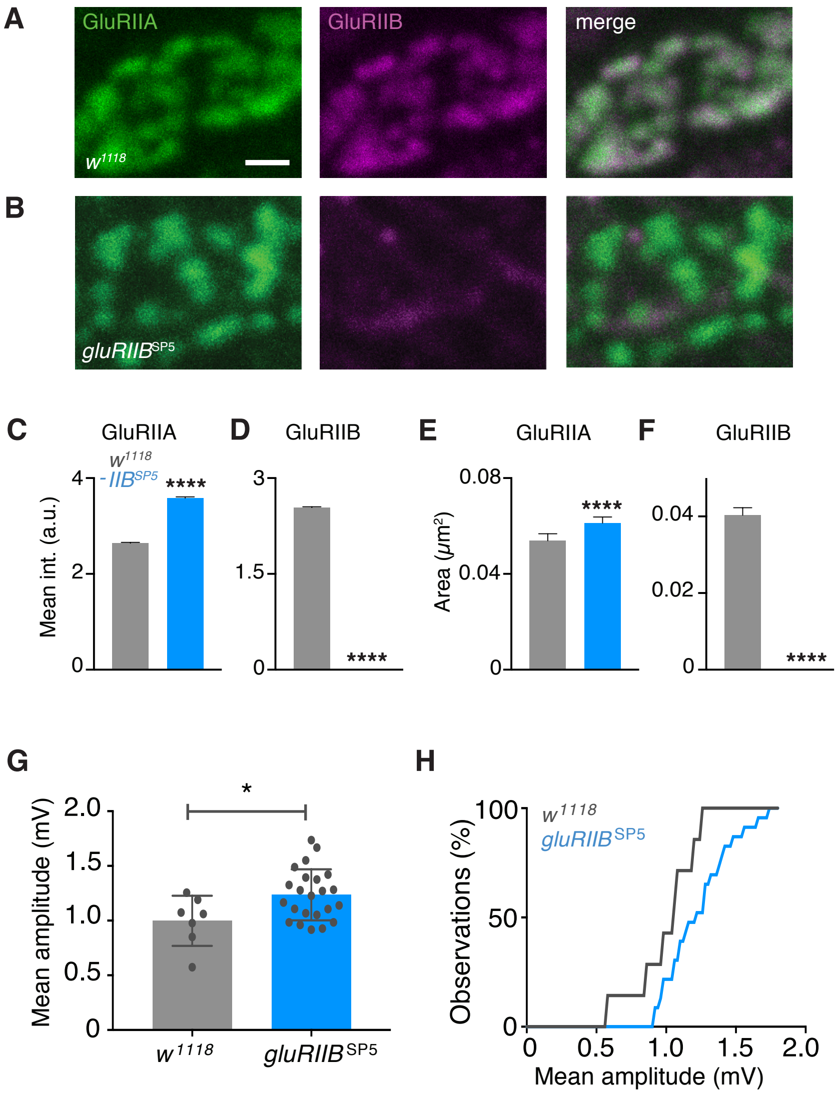

A
GluRIIA
GluRIIB
merge
w1118
B
gluRIIBSP5
C
GluRIIA
w1118
IIBSP5
Mean int. (a.u.)
****
D
GluRIIB
****
E
GluRIIA
Area (μm²)
****
F
GluRIIB
****
G
Mean amplitude (mV)
*
w1118
gluRIIBSP5
H
Observations (%)
w1118
gluRIIBSP5
Mean amplitude (mV)

Fig. S7 GluRIIB-mutant (**A**-**B**) Representative confocal images of *w^{1118}* and *$gluRIIB^{SP5}$* boutons stained with anti-GluRIIA (green) and anti-GluRIIB (magenta). (**C**-**D**) Average mean intensity of GluRIIA and GluRIIB in *w^{1118}* (gray) and *$gluRIIB^{SP5}$* (blue) (C: *w^{1118}*: *n* = 1101; 2.642 a.u. ± 0.017; *$GluRIIB^{SP5}$*: *n* = 980; 3.581 a.u. ± 0.026; *P* < 0.0001; D: *w^{1118}*: *n* = 1384; 2.538 a.u. ± 0.014; *$GluRIIB^{SP5}$*: no anti-GluRIIB staining in the *GluRIIB*-mutant). (**E**-**F**) Area of Brp and GluRIIB in the indicated genotypes (E: *w^{1118}*: *n* = 1101; 0.054 μm^2 ± 0.003; *$GluRIIB^{SP5}$*: *n* = 980; 0.061 μm^2 ± 0.003; *P* < 0.0001; F: *w^{1118}*: *n* = 1384; 0.040 μm^2 ± 0.002; *$GluRIIB^{SP5}$*: no anti-GluRIIB staining in the *GluRIIB*-mutant). **(G)** Average mEPSP mean amplitude of *w^{1118}* (gray) and *gluRIIB*^{SP5} mutants (blue). (**H**) Corresponding cumulative frequency histogram of mean mEPSP amplitude of the indicated genotypes (G-H: *w^{1118}*: *N* = 7; 0.998 mV ± 0.087; *$GluRIIB^{SP5}$*: *N* = 23; 1.236 mV ± 0.049; *P* = 0.037). Scale bar: (A) 1 µm.

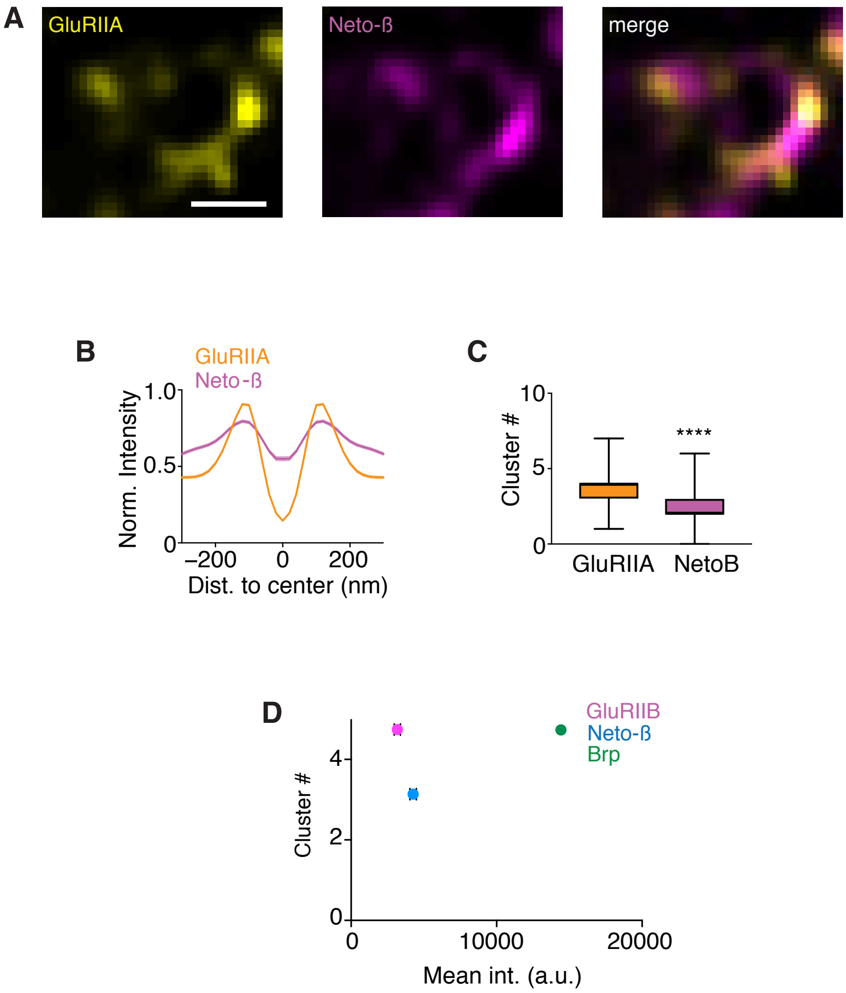
A
GluRIIA
Neto-ß
merge
B
GluRIIA
Neto-ß
Norm. Intensity
1.0
0.5
0
–200
0
200
Dist. to center (nm)
C
Cluster #
10
5
0
****
GluRIIA
NetoB
D
GluRIIB
Neto-ß
Brp
Cluster #
4
2
0
0
10000
20000
Mean int. (a.u.)

Fig. S8. Neto-β rings. (A) High magnification of a max. intensity projection of a representative wild-type ring stained for anti-GluRIIA and anti-Neto-β imaged with gSTED. **(B)** Normalized fluorescence intensity line profiles of the GluRIIA (orange) and the Neto-β channel (magenta) (the shaded area represents s.e.m.). (n = 335; perimeter and center: P < 0.0001) **(C)** Average cluster number (median ± min./max.) within GluRIIA- and Neto-β rings (n-GluRIIA = 311; 3.762 ± 0.067; n-Neto-β = 310; 2.648 ± 0.069; P < 0.0001). (**D**) Cluster number per ring (based on STED images) plotted as function of mean intensity in w^{1118} stained with anti-GluRIIB (magenta), anti-Neto-β (blue) or Brp (green) (based on confocal images); note that there is no correlation between the fluorescence intensity and the number of clusters detected. (GluRIIB: Mean Int.: n = 175; 3177.143 a.u. ± 208.72; Cluster #: n = 175; 4.743 ± 0.101; Neto-β: Mean Int.: n = 270; 4260.063 a.u. ± 219.458; Cluster #: n = 268; 3.138 ± 0.125; Brp: Mean Int.: n = 314; 14425.77 a.u. ± 322.14; Cluster #: n = 4.732 ± 0.057). Scale bar: (A) 200 nm.

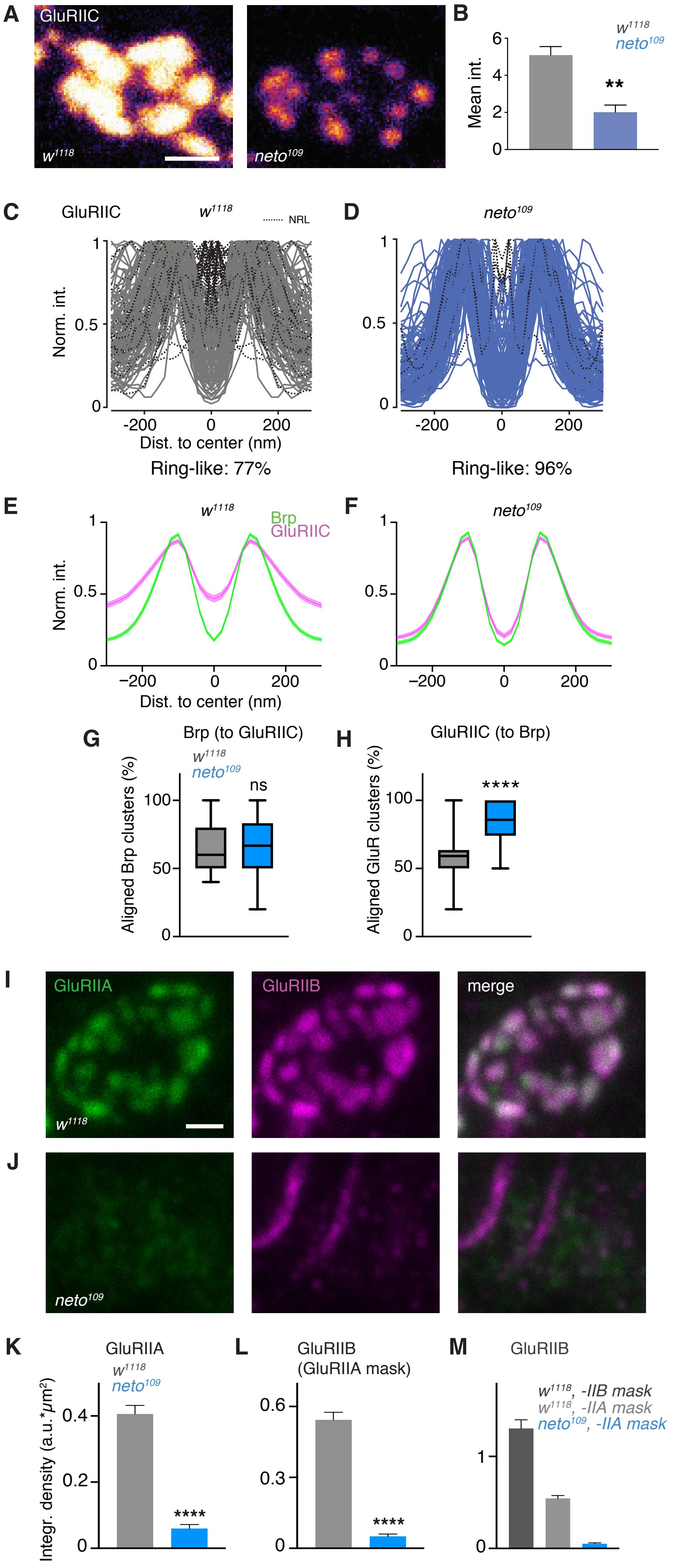

A
GluRIIC
w1118
neto109
B
Mean int.
w1118
neto109
**
C
GluRIIC
w1118
NRL
Norm. int.
Dist. to center (nm)
Ring-like: 77%
D
neto109
Ring-like: 96%
E
w1118
Brp
GluRIIC
Norm. int.
Dist. to center (nm)
F
neto109
G
Brp (to GluRIIC)
w1118
neto109
ns
Aligned Brp clusters (%)
H
GluRIIC (to Brp)
****
Aligned GluR clusters (%)
I
GluRIIA
GluRIIB
merge
w1118
J
neto109
K
GluRIIA
w1118
neto109
Integr. density (a.u.*μm²)
****
L
GluRIIB
(GluRIIA mask)
****
M
GluRIIB
w1118, -IIB mask
w1118, -IIA mask
neto109, -IIA mask

Fig. S9. *neto^{109}* mutant (**A**) Representative max. intensity projection of boutons stained with anti-GluRIIC in *w^{1118}* (left) and *neto*^{109} (right) at confocal resolution (fire LUT). (**B**) Corresponding mean GluRIIC-fluorescence intensity of the indicated genotypes (*N*-*w^{1118}* = 5; 5.096 a.u. ± 0.458; *N*-*neto^{109}* = 5; 2.013 a.u. ± 0.383; *P* = 0.008). (**C**-**D**) Representative GluRIIC-line profiles of a *w^{1118}* NMJ (gray) and a *neto*^{109} mutant NMJ (blue), with all GluRIIC-line profiles detected opposite to the Brp-ring centers (solid lines) and GluRIIC-line profiles with a local maximum at the center and/or a diameter <100 nm (Non-ring like, 'NRL', dashed black lines)'. Note the increased fraction of ring-like profiles in *neto*^{109} mutants (*w^{1118}*: *n* = 132; *n*-NRL = 30.36; *neto^{109}*: *n* = 148; *n*-NRL = 5.92). (**E**-**F**) Brp-GluRIIC channel comparison in *w^{1118}* and *neto^{109}* (*n*-*w^{1118}* = 133; *n*-*neto^{109}* = 149). (**G**) Percentage of Brp clusters aligned to GluRIIC in *w^{1118}* (gray) and *neto^{109}* (blue) (*n*-*w^{1118}* = 30; 65.23% ± 3.091; *n*-*neto^{109}* = 32; 66.41% ± 3.744; *P* = 0.644). (**H**) Percentage of GluRIIC clusters aligned to Brp in *w^{1118}* (gray) and *neto^{109}* (blue) (*n*-*w^{1118}* = 30; 58.21% ± 2.863; *n*-*neto^{109}* = 30; 82.76% ± 3.014; *P* < 0.0001). (**I**-**J**) Representative max. intensity projection of a bouton stained with anti-GluRIIA (green) and anti-GluRIIB (green) in *w^{1118}* (left) and *neto*^{109} (right) at confocal resolution. (**K**-**L**) Integrated densities of GluRIIA and GluRIIB-detected based on the GluRIIA mask in the indicated genotypes (K: *n*-*w^{1118}* = 1007; 0.406 a.u.*μm^2 ± 0.026; *n*-*neto^{109}* = 171; 0.060 a.u.*μm^2 ± 0.012; *P* < 0.0001; L: *n*-*w^{1118}* = 943; 0.543 a.u.*μm^2 ± 0.033; *n*-*neto^{109}* = 159; 0.050 a.u.*μm^2 ± 0.011; *P* < 0.0001). (**M**) Summary of the integrated densities of total GluRIIB in *w^{1118}* and GluRIIB based on the GluRIIA mask in *w^{1118}* (light gray) and *neto^{109}* (blue) (*w^{1118}*: *n*-GluRIIB mask = 1604; 1.304 a.u.*μm^2 ± 0.094; *n*-GluRIIA mask = 943; 0.543 a.u.*μm^2 ± 0.033; *neto*^{109}: *n*-GluRIIA mask = 159; 0.050 a.u.*μm^2 ± 0.011). Scale bars: (A) 1 µm; (I) 1µm.

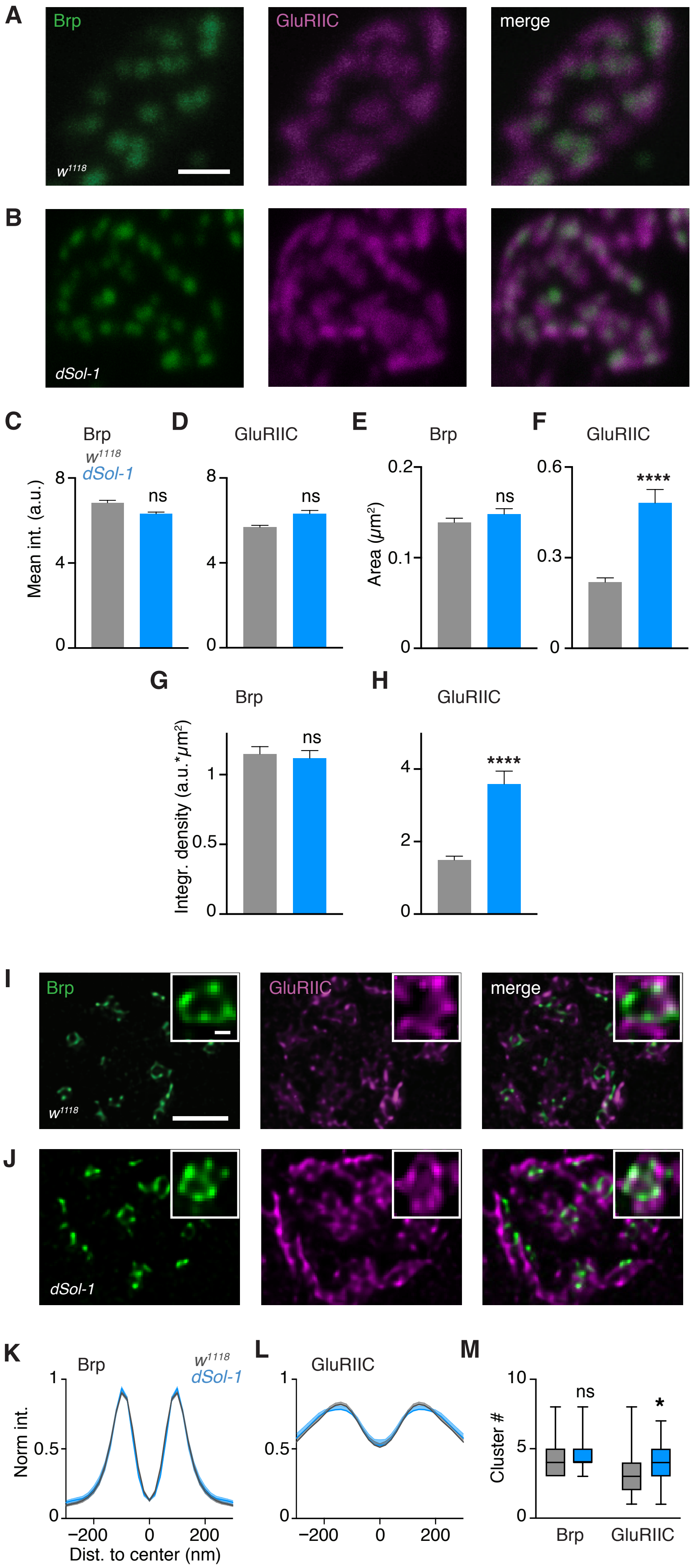
A
Brp
GluRIIC
merge
w1118
B
dSol-1
C
Brp
w1118
dSol-1
Mean int. (a.u.)
ns
D
GluRIIC
ns
E
Brp
Area (μm²)
ns
F
GluRIIC
****
G
Brp
Integr. density (a.u.*μm²)
ns
H
GluRIIC
****
I
Brp
GluRIIC
merge
w1118
J
dSol-1
K
Brp
w1118
dSol-1
Norm int.
Dist. to center (nm)
L
GluRIIC
M
Cluster #
ns
*
Brp
GluRIIC

Fig. S10. *dSol-1* mutant (**A**-**B**) Representative max. intensity projection of boutons stained with anti-Brp (green) and GluRIIC (magenta) in w^{1118} and *dSol-1* at confocal resolution. (**C**-**D**) Average mean intensity of Brp and GluRIIC in w^{1118} (gray) and *dSol-1* (blue) (C: n-w^{1118} = 715; 6.839 a.u. ± 0.111; *n-dSol-1* = 620; 6.317 a.u. ± 0.079; *P* = 0.175; D: n-w^{1118} = 638; 5.681 a.u. ± 0.090; *n-dSol-1* = 427; 6.318 a.u. ± 0.153; *P* = 0.12). (**E**-**F**) Area of Brp and GluRIIC in the indicated genotypes (E: n-w^{1118} = 715; 0.139 μm^2 ± 0.005; *n-dSol-1* = 620; 0.148 μm^2 ± 0.006; *P* = 0.884; F: n-w^{1118} = 638; 0.219 μm^2 ± 0.014; *n-dSol-1* = 427; 0.481 μm^2 ± 0.044; *P* < 0.0001). (**G**-**H**) Integrated density of Brp and GluRIIC in the indicated genotypes (G: n-w^{1118} = 715; 1.149 μm^2 ± 0.051; *n-dSol-1* = 620; 1.118 μm^2 ± 0.055; *P* = 0.41; H: n-w^{1118} = 638; 1.493 μm^2 ± 0.103; *n-dSol-1* = 427; 3.588 μm^2 ± 0.354; *P* < 0.0001). (**I**-**J**) Representative max. intensity projection of Brp (green) and GluRIIC (magenta) rings in w^{1118} and *dSol-1*. (**K**-**M**) Normalized intensity line profiles and average cluster number of Brp and GluRIIC in w^{1118} (gray) and *dSol-1* (blue) (K-L: n-w^{1118} = 110; *n-dSol-1* = 56; M: cluster number: Brp: n-w^{1118} = 110; 4.282 ± 0.1; *n-dSol-1* = 56; 4.393 ± 0.146; *P* > 0.999; GluRIIC: n-w^{1118} = 98; 2.939 ± 0.159; *n-dSol-1* = 55; 3.836 ± 0.181; *P* < 0.0001). Scale bars: (A) 1 µm; (I) 1 µm; (I) inset: 100 nm.

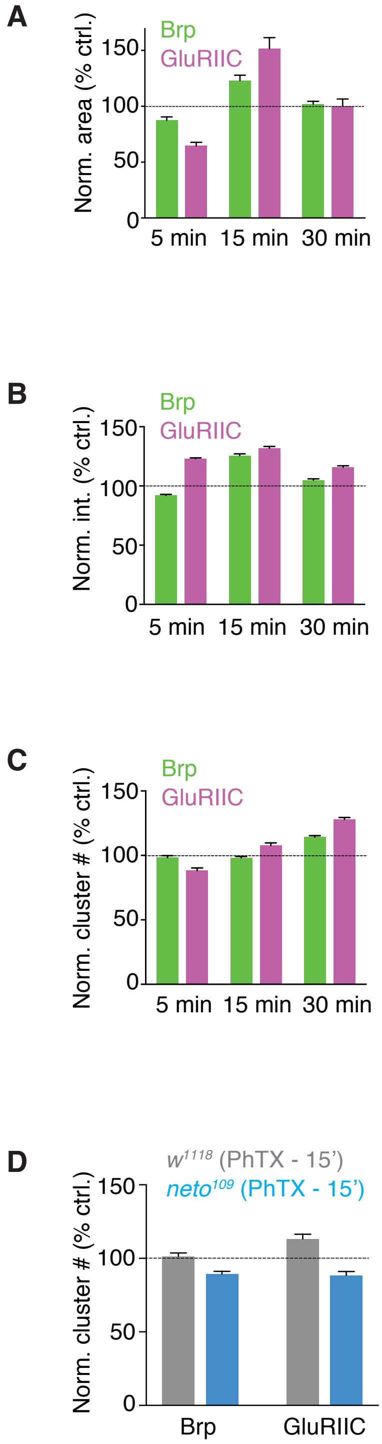
A
Brp
GluRIIC
Norm. area (% ctrl.)
150
100
50
0
5 min
15 min
30 min
B
Brp
GluRIIC
Norm. int. (% ctrl.)
150
100
50
0
5 min
15 min
30 min
C
Brp
GluRIIC
Norm. cluster # (% ctrl.)
150
100
50
0
5 min
15 min
30 min
D
w1118 (PhTX - 15')
neto109 (PhTX - 15')
Norm. cluster # (% ctrl.)
150
100
50
0
Brp
GluRIIC

Fig. S11. Transsynaptic homeostatic modulation of Brp and GluR. **(A, B)** Average Brp- (green) and GluRIIC (magenta) area (*A*) and intensity (*B*) of max. intensity projection confocal data upon PhTX incubation for 5 min, 15 min or 30 min normalized to their respective time-matched HL3 controls (The data shown in *B* are re-plotted from Fig. 4I to facilitate comparison). Note the significant decrease in Brp- and GluRIIC area after 5 min PhTX treatment (*A*), which likely indicates Brp- and GluR redistribution. Mean Brp- and GluRIIC area (*A*), as well as Brp- and GluRIIC intensity (*B*) increase after 15 min PhTX application, indicating increased Brp- and GluR levels. While mean Brp- and GluRIIC intensity (*B*) are still increased after 30 min PhTX incubation, there is no relative change in Brp- and GluRIIC area (*A*), because of an increase in mean Brp- and GluRIIC area without PhTX treatment. This increase could not be quantified relative to the other time points, because the laser intensity needed to be adjusted. Note that data at a given time point were recorded with the same laser intensity and settings. The increase in baseline Brp- and GluRIIC fluorescence intensity in the absence of PhTX treatment is the focus of a different study in preparation (Brp: *n*-5' = 940; 87.47% ± 3.028; $P < 0.0001$; *n*-15' = 844; 122.9% ± 4.866; $P < 0.0001$; *n*-30' = 1736; 100.1% ±6.371; $P = 0.4809$; GluRIIC: *n*-5' = 1282; 64.59% ± 3.121; $P < 0.0001$; *n*-15' = 724; 151.8% ± 9.767; $P < 0.0001$; *n*-30' = 1024; 100.1% ± 6.371; $P = 0.9816$). **(C)** Average cluster number within the Brp- and the GluRIIC rings upon PhTX incubation for 5 min, 15 min or 30 min normalized to their respective time-matched HL3 controls revealed by gSTED imaging (Brp: *n*-5' = 221; 98.30% ± 1.554; $P = 0.276$; *n*-15' = 332; 98.07% ± 1.17; $P = 0.099$; *n*-30' = 705; 114.2% ± 1.18; $P < 0.0001$; GluRIIC: *n*-5' = 219; 88.19% ± 2.107; $P < 0.0001$; *n*-15' = 332; 107.7% ± 2.042; $P = 0.0002$; *n*-30' = 705; 127.9% ± 1.542; $P < 0.0001$). (**D**) Average cluster number within the Brp- and the GluRIIC ring in w^{1118} and $neto^{109}$ upon PhTX incubation for 15 min normalized to their respective time-matched HL3 controls (Brp: n-w^{1118} = 124; 101.4% ± 2.326; $P = 0.5605$; n-$neto^{109}$ = 252; 89.36% ± 1.839; $P < 0.0001$; GluRIIC: n-w^{1118} = 124; 113.2% ± 3.195; $P < 0.0001$; n-$neto^{109}$ = 244; 88.48% ± 2.576; $P < 0.0001$).

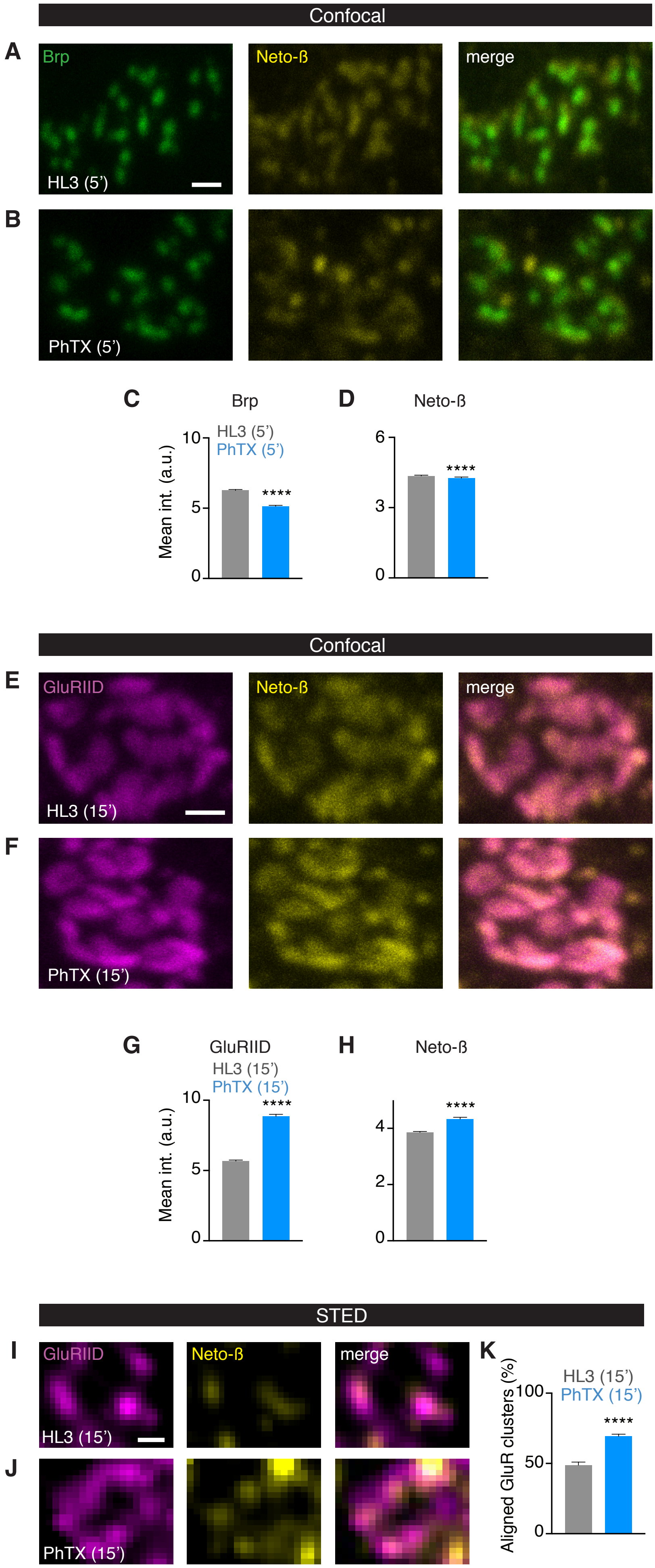
Confocal
A
Brp
Neto-ß
merge
HL3 (5')
B
PhTX (5')
C
Brp
HL3 (5')
PhTX (5')
Mean int. (a.u.)
10
5
0
****
D
Neto-ß
6
3
0
****
Confocal
E
GluRIID
Neto-ß
merge
HL3 (15')
F
PhTX (15')
G
GluRIID
HL3 (15')
PhTX (15')
Mean int. (a.u.)
10
5
0
****
H
Neto-ß
4
2
0
****
STED
I
GluRIID
Neto-ß
merge
HL3 (15')
J
PhTX (15')
K
Aligned GluR clusters (%)
HL3 (15')
PhTX (15')
100
50
0
****

Fig. S12. Neto-β during PHP. (**A**-**B**) Representative w^{1118} boutons stained with Brp (green) and Neto-β (yellow) upon HL3 or PhTX treatment (both 5') at confocal resolution. (**C**-**D**) Average mean intensity of Brp and anti-Neto-β in HL3 (gray) and PhTX (blue) (C: *n*-HL3 = 964; 6.258 a.u. ± 0.068; *n*-PhTX = 837; 5.136 a.u. ± 0.072; *P* 0.0001; D: *n*-HL3 = 2063; 4.359 a.u. ± 0.029; *n*-PhTX = 2071; 4.259 a.u. ± 0.047; *P* < 0.0001). (**E**-**F**) Representative w^{1118} boutons stained with anti-GluRIID (magenta) and Neto-β (yellow) upon HL3 or PhTX treatment (both 15') at confocal resolution. (**G**-**H**) Corresponding average mean intensity of Brp and Neto-β after 15 min of HL3 (gray) and PhTX (blue) treatment (G: *n*-HL3 = 737; 5.673 a.u. ± 0.074; *n*-PhTX = 507; 8.847 a.u. ± 0.151; *P* < 0.0001; H: *n*-HL3 = 748; 3.858 a.u. ± 0.033; *n*-PhTX = 541; 4.327 a.u. ± 0.065; *P* < 0.0001). (**I**-**J**) w^{1118} boutons stained with anti-GluRIID (magenta) and anti-Neto-β (yellow) rings following HL3 or PhTX treatment (both: 15'). (**K**) Percentage of GluRIIC clusters aligned to Brp after treatment with HL3 (gray) or PhTX (blue) (both 15') (*n*-HL3 = 88; 48.8% ± 2.12; *n*-PhTX = 143; 69.31% ± 1.475; *P* < 0.0001). Scale bars: (A) 1 µm; (E) 1µm; (I) 100 nm.
